# Event rates and incidence of post-COVID-19 condition in hospitalised SARS-CoV-2 positive children and young people and controls across different pandemic waves: exposure-stratified prospective cohort study in Moscow (StopCOVID)

**DOI:** 10.1186/s12916-023-03221-x

**Published:** 2024-02-01

**Authors:** Ekaterina Pazukhina, Mikhail Rumyantsev, Dina Baimukhambetova, Elena Bondarenko, Nadezhda Markina, Yasmin El-Taravi, Polina Petrova, Anastasia Ezhova, Margarita Andreeva, Ekaterina Iakovleva, Polina Bobkova, Maria Pikuza, Anastasia Trefilova, Elina Abdeeva, Aysylu Galiautdinova, Yulia Filippova, Anastasiia Bairashevskaia, Aleksandr Zolotarev, Nikolay Bulanov, Audrey DunnGalvin, Anastasia Chernyavskaya, Elena Kondrikova, Anastasia Kolotilina, Svetlana Gadetskaya, Yulia V. Ivanova, Irina Turina, Alina Eremeeva, Ludmila A. Fedorova, Pasquale Comberiati, Diego G. Peroni, Nikita Nekliudov, Jon Genuneit, Luis Felipe Reyes, Caroline L. H. Brackel, Lyudmila Mazankova, Alexandra Miroshina, Elmira Samitova, Svetlana Borzakova, Gail Carson, Louise Sigfrid, Janet T. Scott, Sammie McFarland, Matthew Greenhawt, Danilo Buonsenso, Malcolm G. Semple, John O. Warner, Piero Olliaro, Ismail M. Osmanov, Anatoliy A. Korsunskiy, Daniel Munblit, Sechenov StopCOVID Research Team, Khazhar Aktulaeva, Islamudin Aldanov, Nikol Alekseeva, Ramina Assanova, Asmik Avagyan, Irina Babkova, Lusine Baziyants, Anna Berbenyuk, Tatiana Bezbabicheva, Julia Chayka, Iuliia Cherdantseva, Yana Chervyakova, Tamara Chitanava, Alexander Chubukov, Natalia Degtiareva, Gleb Demyanov, Semen Demyanov, Salima Deunezhewa, Aleksandr Dubinin, Anastasia Dymchishina, Murad Dzhavadov, Leila Edilgireeva, Veronika Filippova, Yuliia Frumkina, Anastasia Gorina, Cyrill Gorlenko, Marat Gripp, Mariia Grosheva, Eliza Gudratova, Elena Iakimenko, Margarita Kalinina, Ekaterina Kharchenko, Anna Kholstinina, Bogdan Kirillov, Herman Kiseljow, Natalya Kogut, Polina Kondrashova, Irina Konova, Mariia Korgunova, Anastasia Kotelnikova, Alexandra Krupina, Anna Kuznetsova, Anastasia Kuznetsova, Anna S. Kuznetsova, Anastasia Laevskaya, Veronika Laukhina, Baina Lavginova, Yulia Levina, Elza Lidjieva, Anastasia Butorina, Juliya Lyaginskaya, Ekaterina Lyubimova, Shamil Magomedov, Daria Mamchich, Rezeda Minazetdinova, Artemii Mingazov, Aigun Mursalova, Daria Nikolaeva, Alexandra Nikolenko, Viacheslav Novikov, Georgiy Novoselov, Ulyana Ovchinnikova, Veronika Palchikova, Kira Papko, Mariia Pavlova, Alexandra Pecherkina, Sofya Permyakova, Erika Porubayeva, Kristina Presnyakova, Maksim Privalov, Alesia Prutkogliadova, Anna Pushkareva, Arina Redya, Anastasia Romanenko, Filipp Roshchin, Diana Salakhova, Maria Sankova, Ilona Sarukhanyan, Viktoriia Savina, Ekaterina Semeniako, Valeriia Seregina, Anna Shapovalova, Khivit Sharbetova, Nataliya Shishkina, Anastasia Shvedova, Valeriia Stener, Valeria Ustyan, Yana Valieva, Maria Varaksina, Katerina Varaksina, Ekaterina Varlamova, Natalia Vlasova, Margarita Yegiyan, Nadezhda Ziskina, Daniella Zolochevskaya, Elena Zuykova

**Affiliations:** 1 Laboratory of Health Economics, Institute of Applied Economic Studies, The Russian Presidential Academy of National Economy and Public Administration, Moscow, Russia; 2 Center for Advanced Financial Planning, Macroeconomic Analysis and Financial Statistics, Financial Research Institute of the Ministry of Finance of the Russian Federation, Moscow, Russia; 3 Department of Paediatrics and Paediatric Infectious Diseases, Institute of Child’s Health, Sechenov First Moscow State Medical University (Sechenov University), Moscow, Russia; 4 Tareev Clinic of Internal Diseases, Sechenov First Moscow State Medical University (Sechenov University), Moscow, Russia; 5 Research and Clinical Center for Neuropsychiatry, Moscow, Russia; 6 Department of Pediatrics, Russian Medical Academy of Continuing Professional Education of the Ministry of Health, Moscow, Russia; 7 School of Applied Psychology, University College Cork, Cork City, Ireland; 8 Department of Paediatrics and Paediatric Rheumatology, Sechenov First Moscow State Medical University (Sechenov University), Moscow, Russia; 9 Department of Clinical and Experimental Medicine, Section of Pediatrics, University of Pisa, Pisa, Italy; 10 Institute for Health Metrics and Evaluation, University of Washington, Seattle, WA USA; 11 Department of PediatricsPediatric Epidemiology, Medical Faculty, Leipzig University, Leipzig, Germany; 12 Universidad de La Sabana, School of Medicine, Chía, Colombia; 13 Pandemic Sciences Institute, University of Oxford, Oxford, UK; 14 Department of Pediatric Pulmonology, Emma Children’s Hospital, Amsterdam University Medical Centers, Amsterdam, the Netherlands; 15 Department of Pediatrics, Tergooi MC, Hilversum, the Netherlands; 16 Sechenov First Moscow State Medical University (Sechenov University), Moscow, Russia; 17 Russian Medical Academy of Continuous Professional Education of the Ministry of Healthcare of the Russian Federation, Moscow, Russia; 18 ZA Bashlyaeva Children’s Municipal Clinical Hospital, Moscow, Russia; 19 Research Institute for Healthcare Organization and Medical Management of Moscow Healthcare Department, Moscow, Russia; 20 ISARIC Global Support Centre, Pandemic Sciences Institute, Nuffield Department of Medicine, University of Oxford, Oxford, UK; 21 MRC-University of Glasgow Centre for Virus Research, Glasgow, UK; 22 Long Covid Kids & Friends Charity, Crowhurst, UK; 23 Department of Pediatrics, Section of Allergy/Immunology, Children’s Hospital Colorado, University of Colorado School of Medicine, Aurora, USA; 24 Department of Woman and Child Health and Public Health, Fondazione Policlinico Universitario A. Gemelli IRCCS, Rome, Italy; 25 Dipartimento Di Scienze Biotecnologiche Di Base, Cliniche Intensivologiche E Perioperatorie, Università Cattolica del Sacro Cuore, Rome, Italy; 26 Center for Global Health Research and Studies, Università Cattolica del Sacro Cuore, Roma, Italy; 27 Health Protection Research Unit in Emerging and Zoonotic Infections, Institute of Infection, Veterinary and Ecological Sciences, Faculty of Health and Life Sciences, University of Liverpool, Liverpool, UK; 28 Department of Respiratory Medicine, Alder Hey Children’s Hospital, Liverpool, UK; 29 Inflammation, Repair and Development Section, National Heart and Lung Institute, Faculty of Medicine, Imperial College London, London, UK; 30 Care for Long Term Conditions Division, Florence Nightingale Faculty of Nursing, Midwifery and Palliative Care, King’s College London, London, UK

**Keywords:** Children, Controlled study, COVID-19, COVID-19 sequelae, Incidence, Long COVID, Post-acute sequelae of SARS-CoV-2 infection, PASC, Post COVID-19 condition

## Abstract

**Background:**

Long-term health outcomes in children and young people (CYP) after COVID-19 infection are not well understood and studies with control groups exposed to other infections are lacking. This study aimed to investigate the incidence of post-COVID-19 condition (PCC) and incomplete recovery in CYP after hospital discharge and compare outcomes between different SARS-CoV-2 variants and non-SARS-CoV-2 infections.

**Methods:**

A prospective exposure-stratified cohort study of individuals under 18 years old in Moscow, Russia. Exposed cohorts were paediatric patients admitted with laboratory-confirmed COVID-19 infection between April 2 and December 11, 2020 (Wuhan variant cohort) and between January 12 and February 19, 2022 (Omicron variant cohort). CYP admitted with respiratory and intestinal infections, but negative lateral flow rapid diagnostic test and PCR-test results for SARS-CoV-2, between January 12 and February 19, 2022, served as unexposed reference cohort. Comparison between the ‘exposed cohorts’ and ‘reference cohort’ was conducted using 1:1 matching by age and sex. Follow-up data were collected via telephone interviews with parents, utilising the long COVID paediatric protocol and survey developed by the International Severe Acute Respiratory and Emerging Infection Consortium (ISARIC). The WHO case definition was used to categorise PCC.

**Results:**

Of 2595 CYP with confirmed COVID-19, 1707 (65.7%) participated in follow-up interviews, with 1183/1707 (69%) included in the final ‘matched’ analysis. The median follow-up time post-discharge was 6.7 months. The incidence of PCC was significantly higher in the Wuhan variant cohort (89.7 cases per 1000 person-months, 95% CI 64.3–120.3) compared to post-infection sequalae in the reference cohort (12.2 cases per 1000 person-months, 95% CI 4.9–21.9), whereas the difference with the Omicron variant cohort and reference cohort was not significant. The Wuhan cohort had higher incidence rates of dermatological, fatigue, gastrointestinal, sensory, and sleep manifestations, as well as behavioural and emotional problems than the reference cohort. The only significant difference between Omicron variant cohort and reference cohort was decreased school attendance. When comparing the Wuhan and Omicron variant cohorts, higher incidence of PCC and event rates of fatigue, decreased physical activity, and deterioration of relationships was observed. The rate of incomplete recovery was also significantly higher in the Wuhan variant cohort than in both the reference and the Omicron variant cohorts.

**Conclusions:**

Wuhan variant exhibited a propensity for inducing a broad spectrum of physical symptoms and emotional behavioural changes, suggesting a pronounced impact on long-term health outcomes. Conversely, the Omicron variant resulted in fewer post-infection effects no different from common seasonal viral illnesses. This may mean that the Omicron variant and subsequent variants might not lead to the same level of long-term health consequences as earlier variants.

**Supplementary Information:**

The online version contains supplementary material available at 10.1186/s12916-023-03221-x.

## Background

Multiple studies have attempted to determine the incidence and risk factors associated with SARS-CoV-2 sequelae in children and young people (CYP) [1]. However, these studies were often impeded by methodological heterogeneity, biases, and inconsistent definitions of COVID-19 consequences [2, 3]. The difficulty of appropriate reference or control group selection has subjected available studies to criticism [4].

One possible approach to obtaining a reference group is to recruit uninfected CYP whose negative status has been previously verified by PCR testing. In the CLoCk study, PCR-positive CYP were matched to test-negative CYP using the national SARS-CoV-2 testing dataset [5]. Similarly, a nationwide cohort study of CYP in Denmark included an exposed group with SARS-CoV-2 infection, verified by RT-PCR, and a reference group of randomly selected individuals who have never been test-positive for SARS-CoV-2 [6]. In a mobile app-based study, Molteni and co-authors recruited a reference group of CYP whose self-reported test results were negative [7]. In other studies, the exposed cohorts were formed from randomly selected schools based on RT-PCR test results, and reference cohorts were recruited from CYP who visited their doctor routinely, with both negative RT-PCR and antibody-based SARS-CoV-2 analysis [8, 9].

Previous efforts suffer from blending symptomatic and asymptomatic CYP in the infected groups, which does not address the possible confounding effect of severity or confounding by factors predisposing to more severe symptoms. Additionally, misdiagnosis may have occurred, as acknowledged by some authors [5]. One approach is to limit the scope of the infected group to those CYP who are symptomatic. However, the results of such studies are challenging to interpret, as previously ill CYP are compared to a generally healthy reference, and the incremental effect cannot be evaluated in the context of other previously known illnesses [10]. Previous expert statements [11, 12] as well as recent systematic review suggested that future studies would benefit from control group and adjustment of the study results for health and environmental factors, including SARS-CoV-2 variant [1].

A potential solution to this problem could be using a reference with comparably severe disease, yet without SARS-CoV-2 infection. To the best of our knowledge, a single study followed this consideration, recruiting reference cohort from both hospitalised and non-hospitalised CYP with other non-SARS-CoV-2 community-acquired infections, clinically and laboratory-confirmed, whereas cases were defined as CYP with previous acute phase of COVID-19 [13]. Given the relatively rare occurrence of highly severe SARS-CoV-2 infection [14], filling the gaps in understanding long consequences of SARS-CoV-2 on health and well-being of CYP in comparison to other known infection is needed. Additionally, there is a paucity of paediatric studies evaluating the long-term consequences of COVID-19 related to the most prevalent circulating variants of SARS-CoV-2 in the population during a specific time period [15].

Another limitation pertains to the broad outcome definition employed by most of the observational studies examining the consequences of COVID-19. Although the World Health Organization (WHO) has provided a definition for the post-COVID-19 Condition [16], a disappointingly small number of studies have chosen to incorporate this definition into their research methodology.

This prospective exposure-stratified cohort study aimed to investigate the incidence of post-COVID-19 condition among hospitalised CYP with COVID-19 infection compared with post-infection sequalae in a reference cohort of previously hospitalised CYP with non-COVID-19 infectious diseases. The study used standardised follow-up data collection protocols developed by the International Severe Acute Respiratory and Emerging Infection Consortium (ISARIC) Global Paediatric COVID-19 follow-up working group. The study included assessment of post-COVID-19 condition corresponding to different waves of the pandemic in Moscow, Russia, as a proxy for infection with different SARS-CoV-2 variants.

## Methods

The Strengthening the Reporting of Observational Studies in Epidemiology (STROBE) checklist for cohort studies (https://www.strobe-statement.org/) was utilised to report the present study.

### Study design, setting, and participants

The present study is an exposure-stratified prospective cohort study of CYP under the age of 18 carried out in Bashlyaeva Children’s City Clinical Hospital and G.N. Speransky Children’s City Clinical Hospital No. 9. These hospitals were the primary hospitals treating COVID-19 in CYP residing in Moscow at different times of pandemic. Additional file 1: Table S1 provides a detailed description of the criteria for hospital admission according to the local clinical guidelines.

Exposed cohorts for this study included paediatric patients hospitalised to Bashlyaeva Children’s City Clinical Hospital with a laboratory-confirmed diagnosis of COVID-19 infection, spanning from April 2, 2020, to December 11, 2020 (Wuhan variant cohort), and to G.N. Speransky Children’s City Clinical Hospital No. 9 between January 12, 2022, and February 19, 2022 (Omicron variant cohort). The dates of patient hospital admission were matched with the data on variant predominance in Moscow [17] (Additional file 1: Figure S1).

CYP who were hospitalised to G.N. Speransky Children’s City Clinical Hospital No. 9 with confirmed respiratory and gastrointestinal infections in conjunction with negative lateral flow rapid diagnostic test and PCR-test results for SARS-CoV-2, between January 12, 2022, and February 19, 2022, served as the unexposed reference cohort for this study. They represent typical patients who are routinely admitted to the hospital with respiratory and gastrointestinal infections during seasonal outbreaks. Recruiting a reference cohort during the Wuhan wave proved to be a formidable challenge. The dearth of admissions of CYP with respiratory infections other than COVID-19, coupled with the high rate of false negatives in COVID-19 testing during the initial months of the pandemic and the scarcity of resources further compounded the difficulty.

### Data management

Baseline data at admission consisted of information pertaining to patient demographics, symptoms, and comorbidities, documented at the time of admission. In addition, the dataset also encompassed results of clinical investigations, supportive care requirements during hospitalisation, and crucial clinical outcomes upon discharge.

Follow-up data have been collected via telephone interviews with parents conducted by research team members who had received standardised training in interview administration, REDCap data entry, and data security [18–20]. The interviews took place between January 31 and February 27, June 2 and August 1, 2021, and August 1 and September 15, 2022. The long COVID paediatric protocol and survey, which was developed by the International Severe Acute Respiratory and Emerging Infection Consortium (ISARIC) and translated into Russian, was utilised to collect data from both exposed and the reference cohorts. The data included questions regarding symptoms and their onset and persistence at the time of the follow-up as well as the physical, psychosocial, and behavioural well-being of CYP (all proxy-reported by parents).

Data collection, storage, and management were performed using REDCap electronic data capture tools (Vanderbilt University, Nashville, TN, USA) hosted at Sechenov University and Microsoft Excel (Microsoft Corp, Redmond, WA, USA).

### Definitions

The present study adopts the WHO case definition to categorise post-COVID-19 condition as the presence of any symptom that emerges no later than 3 months post-hospital discharge and persists for at least 2 months. In the absence of reliable objective medical record data pertaining to the date of first symptom appearance, symptom duration was calculated from the time of hospital discharge.

Post-COVID-19 condition/post-infection sequalae symptoms were categorised into nine manifestations, which encompassed cardiovascular, dermatological, fatigue, gastrointestinal, musculoskeletal, neurocognitive, respiratory, sensory, and sleep-related symptoms. This classification was formulated by drawing upon relevant prior literature and through ISARIC’s working group deliberations [20].

Emotional and behavioural changes, which were initially recorded using a five-point scale ranging from ‘much less’ to ‘much more’, have been converted into binary variables. We considered worsening of symptoms for excessive fatigue when parents were reporting ‘more’ and ‘much more’ with regard to this outcome during telephone interview.

Recovery was assessed using a Likert scale ranging from ‘1’ (no recovery) to ‘10’ (complete recovery). Responses were transformed into a binary variable, with a ‘1–6’ considered ‘incomplete recovery’ and ‘7–10’ complete recovery from the infection.

Patients in all the cohorts were defined as severe if required non-invasive ventilation, invasive ventilation, or intensive care unit (ICU) care during acute phase of COVID-19.

### Statistical analysis

Baseline characteristics were analysed through descriptive statistics. We summarised continuous variables as medians (interquartile range, IQR), while categorical variables were presented as frequencies (percentages).

We utilised forest plots to depict the incidence and event rates associated with post-COVID-19 conditions and their varying manifestations. The incidence was calculated as the ratio of the total number of new cases to the total time at risk. In line with the World Health Organization’s definition of post-COVID-19 condition (PCC), an individual’s time at risk is capped at three months from the onset of symptoms (or from hospital admission if symptom onset is unknown). For manifestations of symptoms, we selected the shortest time at risk across all symptoms for each individual within a group. We calculated the rate of events as the ratio of the number of cases to the number of people at risk.

We conducted a comparison between the ‘exposed cohorts’ (Wuhan variant cohort and Omicron variant cohort) and ‘reference cohort’ using 1:1 matching by age (± 1 year) and sex. In a sensitivity analysis, two additional matching parameters severity and length of hospitalisation (± 2 days) were included (Additional file 1: Table S2, Figures S2 and S3). We regarded age and sex as confounders, while severity (defined as the need for non-invasive or invasive ventilation, or intensive care unit admission) and length of hospitalisation may function as proxies for true severity.

We employed bootstrap methodology (30,000 iterations) to obtain 95% confidence intervals (CIs) for the estimates of post-COVID-19 condition event rates and incidence. This involved resampling from a variety of potentially matched cohorts. We have presented the demographic characteristics and comorbidities of the matched cohorts, alongside the features of the initial sample.

We considered differences in event rates and incidence of manifestations between groups as significant if the median *p*-value for rate ratios, acquired from the bootstrap procedure via the exact Fisher’s test, was less than 5%.

Statistical analysis was conducted using R version 4.0.2, employing the dplyr, foreign, ggraph, and ggforce libraries.

To minimise recall bias, we limited the period from the onset date of the first symptoms (or the date of hospital admission if symptom onset was missing or inconsistent) to the follow-up date to a maximum of 8 months.

## Results

### Study participant characteristics

**Fig. 1:**
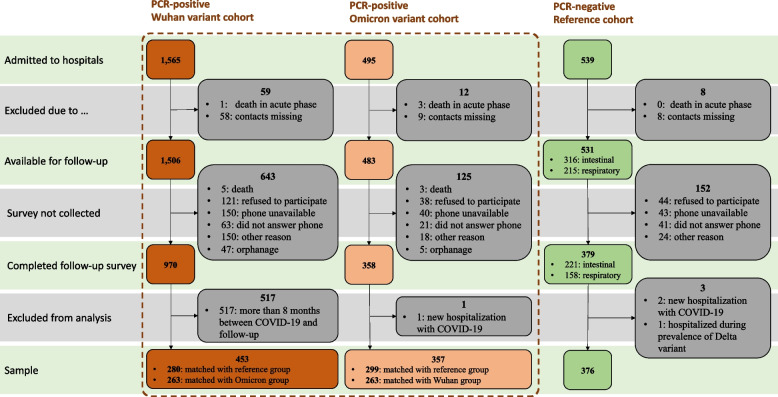
Study flow chart

Out of the 2595 eligible CYP with laboratory-confirmed COVID-19 who were discharged between March 18, 2020, and February 20, 2022, 2520 (97.1%) had contact information available. Of these, 1707 CYP (65.7% of those discharged, 67.7% of those with contact information) participated in follow-up interviews. Upon matching, 1183 out of 1707 CYP (69%) were included in the final analysis (Fig. 1).

Table 1 presents the demographic and clinical characteristics of the study participants. The median time elapsed after hospital discharge was 6.7 months, ranging from 6.2 months for Omicron variant and control cohorts to 7.5 months in the Wuhan variant cohort. Before the matching process, the median age was 10.3 years (IQR 2.8–14.7) in the Wuhan variant cohort, 2.6 years (IQR 0.8–7.0) in the Omicron stain cohort, and 4.2 years (IQR 1.9–7.8) in the reference cohort. The share of severe patients ranged from 2.4% in the Wuhan variant cohorts to 6.2% in the Omicron cohort and 3.2% in the reference cohort.

**Table 1:** Demographic characteristics of study participants

Characteristic	Initial sample			Matched Wuhan-Reference		Matched Omicron-Reference		Matched Wuhan-Omicron	
	Wuhan variant cohort	Omicron variant cohort	Reference cohort	Wuhan variant cohort	Reference cohort	Omicron variant cohort	Reference cohort	Wuhan variant cohort	Omicron variant cohort
Number of participants	453	357	376	279	279	299	299	263	263
Median time from the hospital discharge to the follow-up point (IQR), months	7.5 (7.3, 7.7)/453	6.2 (6.1, 6.3)/357	6.2 (6.1, 6.4)/376	7.5 (7.3–7.7)	6.2 (6.1–6.3)	6.1 (6.1–6.3)	6.2 (6.1–6.3)	7.5 (7.3–7.7)	6.2 (6.1–6.3)
Length of hospitalisation, days	7.0 (4.0, 10.0)/453	3.3 (1.8, 5.5)/357	3.2 (2.0, 5.0)/375	7.0 (4.0–9.0)	3.3 (2.1–5.3)	3.1 (1.7–5.3)	3.1 (2.0–5.0)	7.0 (4.0–10.0)	3.3 (1.8–5.6)
Gender, female	239/453 (53%)	164/357 (46%)	190/376 (51%)	48.4%	48.4%	50.7%	50.7%	51.7%	51.7%
Median age (IQR) at hospital admission, years	10.3 (2.8, 14.7)/453	2.6 (0.8, 7.0)/357	4.2 (1.9, 7.8)/376	3.9 (1.2–9.5)	4.2 (1.5–9.4)	3.3 (1.1–7.6)	3.4 (1.6–7.4)	3.6 (1.0–9.1)	3.4 (1.0–8.9)
Acute COVID-19 severity	11/453 (2.4%)	22/357 (6.2%)	11/376 (2.9%)	1.8%	2.7%	6.7%	3.0%	1.9%	6.8%
Comorbidities									
Neurological	46/440 (10%)	36/354 (10%)	20/375 (5.3%)	10.8%	5.0%	10.7%	5.3%	10.6%	10.8%
Neurodisability	13/438 (3.0%)	16/353 (4.5%)	6/373 (1.6%)	3.4%	1.4%	4.7%	1.4%	3.2%	5.0%
Heart diseases	22//440 (5.0%)	14/353 (4.0%)	16/373 (4.3%)	4.1%	4.0%	4.0%	4.3%	4.3%	3.9%
Respiratory diseases (not including asthma)	16/441 (3.6%)	13/354 (3.7%)	5/372 (1.3%)	3.7%	1.1%	3.7%	1.3%	2.7%	3.8%
Tuberculosis	0/353 (0%)	0/354 (0%)	0/373 (0%)	0.0%	0.0%	0.0%	0.0%	0.0%	0.0%
Asthma (physician diagnosed)	10/441 (2.3%)	2/353 (0.6%)	8/372 (2.2%)	1.1%	2.5%	0.3%	2.0%	0.4%	0.4%
Allergic rhinitis/hay fever	44/440 (10%)	23/355 (6.5%)	25/373 (6.7%)	8.1%	6.2%	6.1%	6.1%	7.4%	6.2%
Food allergy	69/440 (16%)	28/351 (8.0%)	43/372 (12%)	16.0%	11.3%	8.2%	11.2%	16.4%	8.1%
Atopic dermatitis/Eczema	45/441 (10%)	5/354 (1.4%)	42/374 (11%)	10.2%	11.3%	1.7%	11.4%	10.2%	1.2%
Dermatological problems	22/441 (5.0%)	3/353 (0.8%)	6/372 (1.6%)	3.3%	1.8%	0.7%	1.7%	3.1%	1.1%
Gastrointestinal (gut) problems	56/441 (13%)	25/353 (7.1%)	27/373 (7.2%)	11.4%	8.0%	7.2%	6.4%	11.8%	7.6%
Haematological conditions	5/441 (1.1%)	6/353 (1.7%)	4/373 (1.1%)	0.7%	1.1%	2.0%	1.3%	0.8%	1.5%
Malignancy	1/441 (0.2%)	2/352 (0.6%)	1/374 (0.3%)	0.0%	0.4%	0.7%	0.3%	0.0%	0.8%
Immunity problems	3/441 (0.7%)	6/353 (1.7%)	1/374 (0.3%)	0.7%	0.4%	1.7%	0.0%	0.8%	1.9%
Genetic	9/441 (2.0%)	8/351 (2.3%)	3/373 (0.8%)	3.0%	0.7%	2.4%	0.7%	2.8%	2.3%
Endocrinological conditions	14/441 (3.2%)	2/353 (0.6%)	2/374 (0.5%)	2.2%	0.7%	0.7%	0.3%	2.4%	0.4%
Kidney disease	28/440 (6.4%)	10/352 (2.8%)	14/374 (3.7%)	5.2%	4.0%	3.1%	3.7%	5.8%	3.5%
Overweight and obesity (as defined by clinical staff)	14/441 (3.2%)	1/354 (0.3%)	7/374 (1.9%)	1.9%	2.2%	0.3%	1.7%	1.6%	0.4%
Malnutrition	10/440 (2.3%)	8/354 (2.3%)	20/374 (5.3%)	1.1%	5.7%	2.3%	5.0%	0.8%	2.3%
Rheumatologic disorder	9/439 (2.1%)	1/353 (0.3%)	1/374 (0.3%)	1.9%	0.4%	0.3%	0.3%	1.6%	0.4%
HIV	0/440 (0%)	0/350 (0%)	0/371 (0%)	0.0%	0.0%	0.0%	0.0%	0.0%	0.0%

Median (25%, 75%)/*N*; *n* (%); *n*/*N* (%). Severe acute COVID-19 was defined as requiring non-invasive ventilation or invasive ventilation or ICU

Pre-existing comorbidities and demographic characteristics of the initial cohorts and the matched cohorts are presented in the table. Information regarding pre-existing comorbidities was recorded for patients during hospital admission during acute phase of COVID-19 infection. Data are presented as *n* (%) or median (IQR) excluding missing values. *ICU*, intensive care unit

The most common comorbidities in the pooled initial cohorts were food allergy (12.0%), intestinal (9.3%) and neurological (8.7%) problems, and atopic dermatitis/eczema (7.9%). However, in the matched sets, the prevalence of these comorbidities varied significantly. Higher prevalence of neurological problems (5.8%), food allergy (4.7%), and lower prevalence of malnutrition (4.6%) were observed in Wuhan variant cohort when compared with the reference cohort. Higher prevalence of atopic dermatitis/eczema (9.7%) and neurological problems (+ 5.4%) was found in Omicron variant cohort when compared with the reference cohort.

### Post-COVID-19 condition/post-infection sequalae incidence

**Fig. 2:**
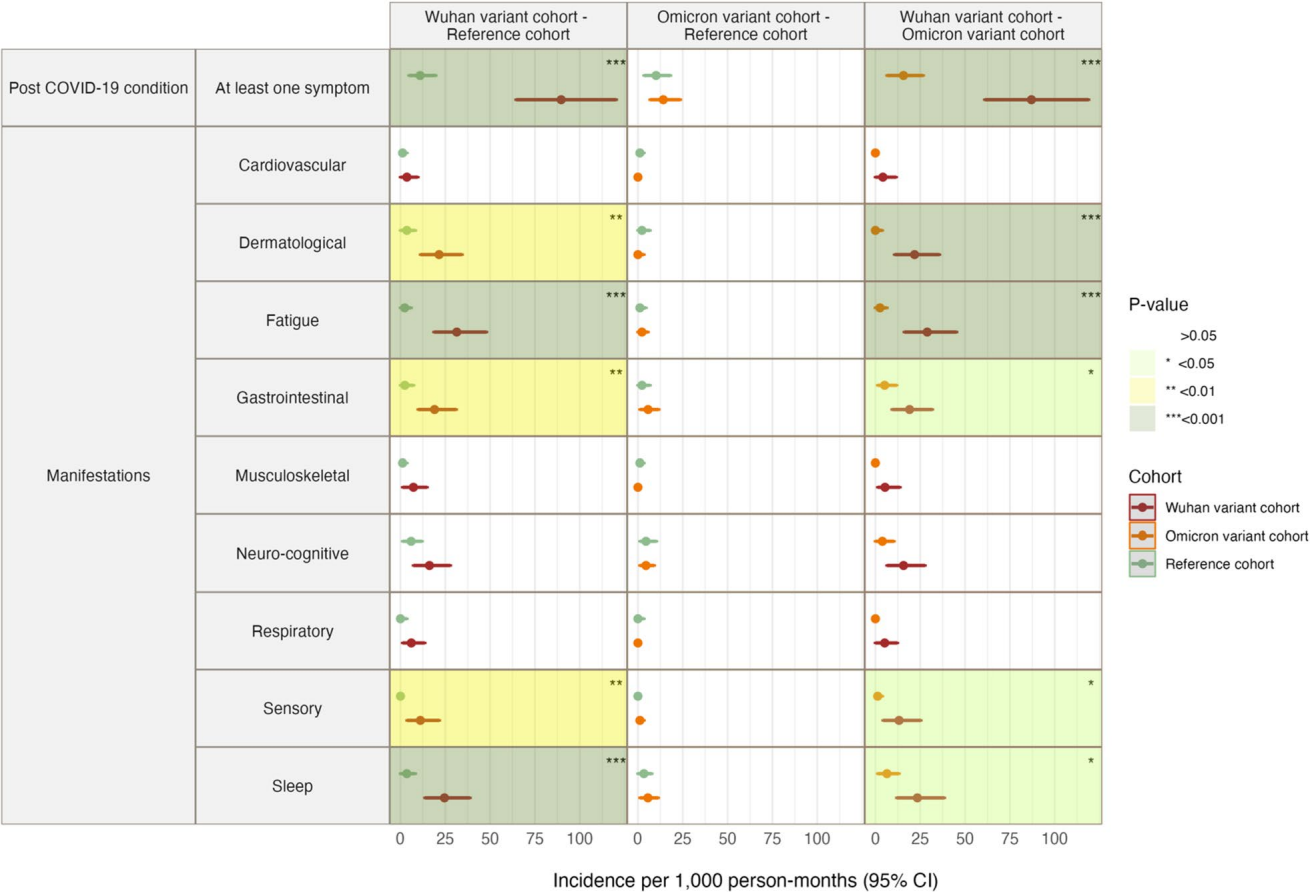
Incidence of post-COVID-19 condition manifestations in Wuhan variant cohort, Omicron variant cohort, and post-infection sequalae manifestations in reference cohort

**Fig. 3:**
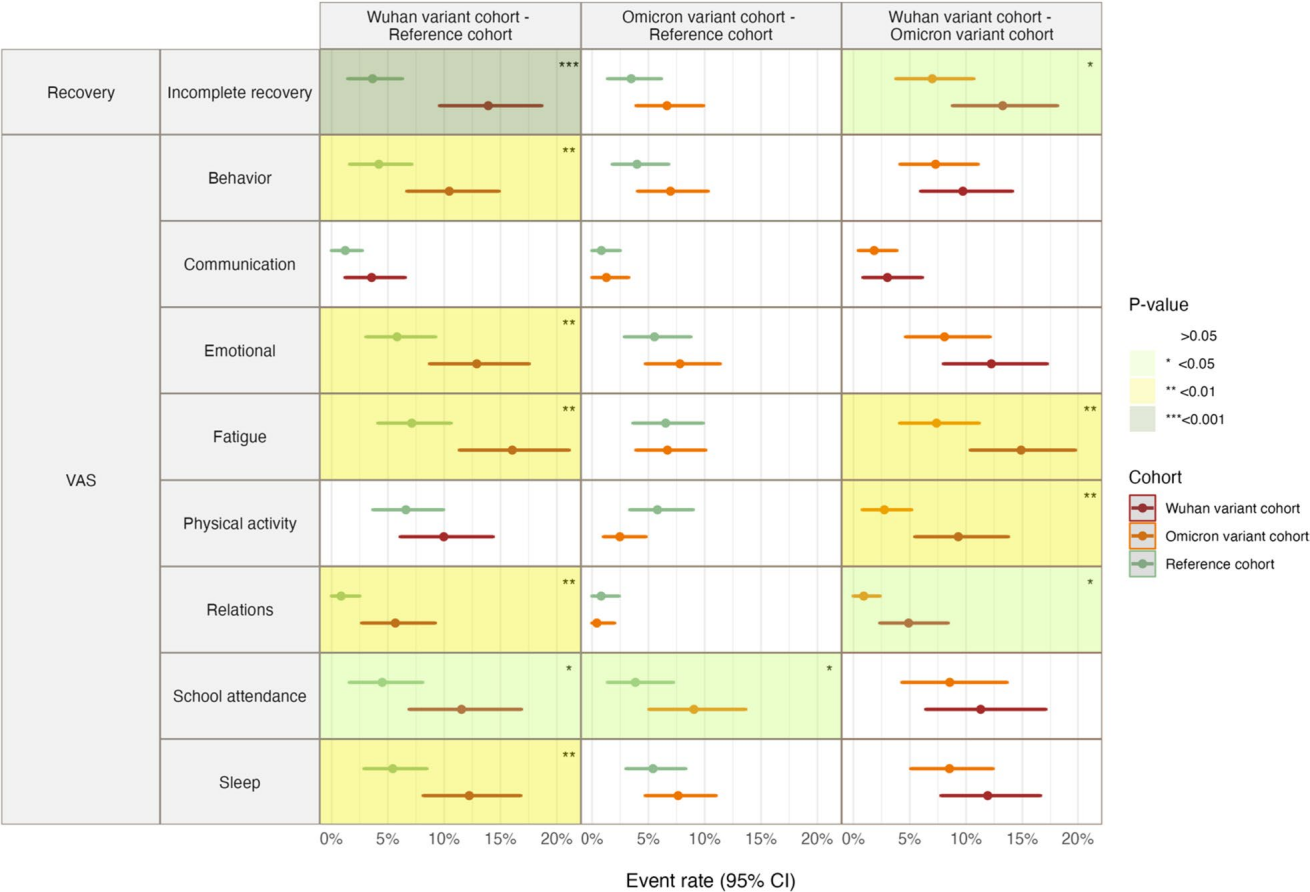
Event rate for incomplete recovery and emotional and behavioural changes in Wuhan variant cohort, Omicron variant cohort, and post-infection sequalae manifestations in reference cohort. VAS, visual analogue scale

Figure 2 presents the incidence rates of PCC/post-infection sequalae and manifestations of symptoms in matched cohorts, while Fig. 3 depicts the well-being assessment results and incomplete recovery in matched cohorts. Detailed information is available in Table S3.

For the Wuhan variant cohort, the incidence of PCC was 89.6 cases per 1000 person-months (95% CI 64.4–120.4), compared to 11.0 cases of post-infection sequalae per 1000 person-months (95% CI 4.8–19.7) in the reference cohort (*p* < 0.001). Conversely, the difference between the matched Omicron variant cohort (14.1 cases per 1000 person-months, 95% CI 6.8–23.7) and the reference cohort (10.1 cases per 1000 person-months, 95% CI 3.4–18.2) was not statistically significant (*p* = 0.45).

When comparing the Wuhan variant cohort (87.0 cases per 1000 person-months, 95% CI 61.0–118.9) to the Omicron variant cohort (15.7 cases per 1000 person-months, 95% CI 6.5–26.7), a significant difference in the PCC event rate was noted (*p* < 0.001).

### Post-COVID-19 condition/post-infection sequalae manifestations

The Wuhan variant cohort displayed a significantly higher incidence of dermatological, fatigue, gastrointestinal, sensory, and sleep manifestations of PCC than similar post-infection sequalae manifestations in the reference cohort. The largest difference in manifestation rates was observed for fatigue (31.4 cases per 1000 person-months, 95% CI 18.7–48.0 vs. 2.4 cases per 1000 person-months, 95% CI 0.0–6.0) and sleep problems (24.5 cases per 1000 person-months, 95% CI 13.6–38.8 vs. 3.6 cases per 1000 person-months, 95% CI 0.0–8.4).

For the Omicron variant cohort, no statistically significant differences in any symptom manifestations were found when compared with the reference cohort.

Comparing the Wuhan and Omicron variant cohorts, significant differences in incidence were observed for dermatological, fatigue, gastrointestinal, sensory and sleep manifestations. The largest differences were observed in incidence of dermatological manifestations in Wuhan vs Omicron variant cohorts (21.9 cases per 1000 person-months, 95% CI 10.7–35.8 vs. 0.0 cases per 1,000 person-months, 95% CI 0.0–3.8) and fatigue (28.9 cases per 1,000 person-months, 95% CI 16.1–45.3 vs. 2.5 cases per 1,000 person-months, 95% CI 0.0–6.5).

The rate of events for incomplete recovery was significantly higher in the Wuhan variant cohort (13.9%, 95% CI 9.6–18.7%) than in the reference cohort (3.6%, 95% CI 1.4–6.3%). For the Omicron variant cohort, this rate was not significantly different from the reference cohort, but it was significantly lower than the Wuhan variant cohort (7.0% (95% CI 3.8–10.7%) vs. 13.3% (95% CI 8.8–18.2%)) (Additional file 1: Table S3).

### Post-COVID-19 condition/post-infection sequalae emotional and behavioural status

Emotional and behavioural changes also differed significantly between the Wuhan variant and reference cohorts, with higher rates observed in the Wuhan variant cohort for behavioural changes (10.4%, 95% CI 6.7–14.9 vs. 4.2%, 95% CI 1.6–7.1%), emotional problems (12.9%, 95% CI 8.7–17.5% vs. 5.8%, 95% CI 3.1–9.2%), fatigue (16.0%, 95% CI 11.4–21.1% vs. 7.1%, 95% CI 4.1–10.6%), communicating with friends personally (5.4%, 95% CI 2.2–9.4% vs 1.4%, 95% CI 0.0–3.3%), worsening relationships (5.7%, 95% CI 2.7–9.2% vs. 0.8%, 95% CI 0.0–2.5%), decline in school attendance (11.5%, 95% CI 6.9–16.8% vs. 4.5%, 95% CI 1.6–8.1%), and sleep problems (12.2%, 95% CI 8.1–16.8% vs. 5.4%, 95% CI 2.9–8.5%).

In contrast, the Omicron variant cohort only showed a significant difference from the reference cohort with regard to lower school attendance (9%, 95% CI 5.1–13.6% vs. 3.8%, 95% CI 1.4–7.2%).

When the Wuhan and Omicron variant cohorts were compared, significant differences in rates of events for emotional behavioural changes were observed for fatigue (14.9%, 95% CI 10.4–19.8% vs. 7.4%, 95% CI 4.1–11.2%), decreased physical activity (9.3%, 95% CI 5.5–13.8% vs. 2.7%, 95% CI 0.8–5.2%), and deterioration of relationships (4.9%, 95% CI 2.4–8.4% vs. 0.9%, 95% CI 0.0–2.4%).

Incidence (per 1000 person-months (95% confidence interval)) of post COVID-19 condition and different manifestations in exposure and reference groups were matched by age and sex. Statistically insignificant results are presented in white; statistically significant difference of *p* < 0.05 between the groups is highlighted in light green; statistically significant difference of *p* < 0.01 between the groups is highlighted in light yellow; statistically significant difference of *p* < 0.001 between the groups is highlighted in light grey.

Prevalence of incomplete recovery is presented as event rate (95% confidence interval). Statistically insignificant results are presented in white; statistically significant difference of *p* < 0.05 between the groups is highlighted in light green; statistically significant difference of *p* < 0.01 between the groups is highlighted in light yellow; statistically significant difference of *p* < 0.001 between the groups is highlighted in light grey.

## Discussion

The importance of investigating the impact of COVID-19 on CYP has been previously emphasised, particularly in light of new variants of the virus circulating in the population [12]. However, current research on the long-term effects of COVID-19 in CYP is limited and is associated with methodological limitations, making it difficult to distinguish between the effects of the virus and the impact of social restrictions [21]. There is also a lack of studies that compare the effects of COVID-19 in CYP with a control group of individuals following other viral infections. Experts previously highlighted the importance of a control group in studies following SARS-CoV-2 infection in CYP, using standardised case definitions [4]. This study aimed to address these gaps by estimating the incidence of PCC and the impact on well-being among CYP and young people, comparing COVID-19 from different variants with a control cohort of CYP hospitalised with non-COVID-19 infections. We found that CYP hospitalised during Wuhan variant wave had a significantly higher incidence of PCC and incomplete recovery than incidence of post-infection sequalae in the controls. Emotional and behavioural changes were also observed in Wuhan cases, including behaviour changes, emotional problems, excessive fatigue, worsening of relationships, decline in school attendance, and problems with sleep. In contrast, CYP admitted to the hospital with COVID-19 during the Omicron variant wave did not differ significantly from CYP hospitalised with respiratory or gastrointestinal infections in terms of long-lasting outcomes, symptom manifestation, and recovery. However, they were shown to have distinctively more problems with school attendance.

The incidence of PCC was significantly higher in the cohort infected by the Wuhan variant compared to the post-infection sequalae incidence in reference cohort, with a disparity of 77.5 cases per 1000 person-months. Conversely, the incidence rate in the Omicron variant cohort did not show a statistically significant difference from the reference cohort. The striking difference between the Wuhan and Omicron cohorts indicates the potential difference in virulence and resultant clinical outcomes associated with the distinct viral variants. Results are somehow reassuring as Wuhan variant is very uncommon in population now, and findings allow to hypothesise that Omicron variant as well as SARS-CoV-2 circulating in the population, originating from Omicron [22], is unlikely to differ in terms of consequences compared with common seasonal viruses significantly. Such discrepancy in long-term outcomes between different variants might be attributable to the variations in the viral structure, particularly in the spike protein [23], which is known to affect the virus’s ability to bind to and enter host cells. Future studies focusing on the specific biological mechanisms behind these differences could provide more definitive answers.

When assessing symptom manifestations of PCC, the Wuhan cohort presented a markedly higher incidence of dermatological, fatigue, gastrointestinal, sensory, and sleep disturbances than the post-infection sequalae manifestations in reference cohort. Among these, fatigue and sleep problems demonstrated the largest difference in manifestation rates. Such findings corroborate the existing literature, where fatigue and sleep issues have been consistently reported as prominent manifestations of PCC, particularly in the context of the Wuhan variant. In contrast, the Omicron cohort showed no significant difference in symptom manifestations compared to the reference cohort, further highlighting the potential differential pathogenicity of the viral variants. The largest differences between the Wuhan and Omicron cohorts were found for dermatological manifestations and fatigue, again underscoring the burden of these symptoms in CYP infected with the Wuhan variant. In contrast, the Omicron cohort showed no significant difference in symptom manifestations compared to the reference cohort, further highlighting the potential differential pathogenicity of the viral variants. The largest differences between the Wuhan and Omicron cohorts were found for dermatological manifestations and fatigue, again underscoring the burden of these symptoms in CYP infected with the Wuhan variant.

Our findings also revealed that CYP infected with the Wuhan variant had significantly higher rates of incomplete recovery and emotional-behavioural changes, such as behavioural changes, emotional problems, fatigue, worsening relationships, decline in school attendance, and sleep problems, as compared to the reference cohort. These results suggest the long-lasting and pervasive impact of COVID-19 on health and well-being of CYP, especially those infected by the Wuhan variant. It is crucial for healthcare providers to recognise these long-term sequelae and to develop appropriate strategies for ongoing support and management.

The Omicron cohort showed a significant difference from the reference cohort only in terms of lower school attendance. This difference could be attributed to factors such as more conservative return-to-school policies during the Omicron wave. Additionally, parental concerns about their child’s health after infection could have contributed to lower school attendance. However, it does imply a potential impact on the children’s educational attainment and warrants further exploration.

One of the major strengths of this study is the inclusion of a control group of CYP who were admitted to the hospital with non-COVID-19 infections. This allows us to estimate the relative burden of long COVID-19 and to compare the outcomes of CYP with COVID-19 infection to those of CYP with other infections. Both cases and controls in this study were exposed to infection-related hospitalisations, which reduces the potential for bias due to differences in health-seeking behaviour. Another strength of the study is the distinction made between the early Wuhan variant and the later Omicron variant of the virus. By comparing the outcomes of CYP infected with these two variants, we are able to explore the differences in the long-term effects of the two variants of the virus on children’s health. The study utilised the International Severe Acute Respiratory and Emerging Infection Consortium (ISARIC) long-term follow-up study case report forms (CRFs) for CYP. This standardised data collection method ensures that the data collected are consistent and comparable across different study sites. In addition, the study used the WHO post-COVID-19 condition definition, which provides a standardised definition of the condition that allows for more accurate and consistent identification of cases.

However, the study also has several limitations. First, all the cohorts represent hospitalised population which do not allow to extrapolate findings on all CYP. The follow-up period has been restricted to 8 months after hospital discharge. This may not be sufficient to capture the full extent of long-term effects of COVID-19 in CYP. Second, the study is limited in its ability to control for extrinsic factors and residual confounding, such as vaccinations and other viral infections, that may have affected the study outcomes. Third, the criteria for hospital admission may have changed over time, and cases across waves can have different acute severity. Fourth, the chronological association of the waves of study with COVID-19 variants was established without laboratory confirmation, and relied upon available open data sources, which may have introduced bias into the study. No patients were recruited during Delta variant wave. Fifth, the study relied on proxy-reported responses from the parents for the follow-up interviews, which may be affected by recall bias and non-blindness of respondents. Additionally, parental bias towards the health of their CYP may have influenced the accuracy of the data collected. Finally, there is a risk of potential selection bias, with those with symptoms more likely to agree to the survey and thus potentially overestimating the prevalence of post-COVID-19 condition. At the onset of the COVID-19 pandemic, when the Wuhan variant was prevalent in Russia, the only facility in Moscow equipped for the hospitalisation of infected children was the Bashlyaeva Children’s City Clinical Hospital. As the pandemic evolved and other variants, including Omicron, became dominant, the G.N. Speransky Children’s City Clinical Hospital No. 9 COVID-19 wards were set-up in response to increasingly large number of patients expected as a part of this wave. Consequently, it was not possible and feasible to gather data from just one hospital. However, given that both hospitals used identical data collection methodologies, the batch effect in the data is unlikely.

## Conclusions

Overall, the findings of this study underscore the importance of continued monitoring and support for CYP who have been hospitalised with COVID-19 infection as well as for those who may be experiencing long-term effects of the virus. Future studies should aim to elucidate the underlying biological mechanisms behind the observed differences in clinical outcomes associated with different viral variants. The results offer some reassurance, given that the Wuhan variant is now quite rare in the population. These findings permit us to conjecture that the Omicron variant, as well as the SARS-CoV-2 variants currently circulating, is unlikely to diverge significantly from common seasonal viruses in terms of their consequences. Policymakers, healthcare providers, and families must work together to ensure that CYP are provided with the resources and support they need to recover fully and to continue to thrive despite the challenges posed by the COVID-19 pandemic.

### Supplementary Information

**Additional file 1: Table S1.** Criteria for hospital admission as per local clinical guidelines. **Table S2.** Demographic characteristics of study participants—initial and matched cases, sensitivity analysis. **Table S3.** Incidence and prevalence of Post COVID-19 manifestations. **Figure S1.** Time of study participants hospital admission and their chronological correspondence to COVID-19 variant dominance in Moscow city. **Figure S2.** Incidence of post-COVID-19 condition manifestations in matched exposed and reference groups, sensitivity analysis. **Figure S3.** Rates of events for incomplete recovery and emotional behavioural changes in matched exposed and reference groups, sensitivity analysis.

## Data Availability

The data that support the findings of this study are available from the corresponding author, DM, upon reasonable request.

## Abbreviations

CI: Confidence interval
CLoCk: Children and young people with long COVID study
COVID-19: Coronavirus disease 2019
CRF: Case report form
CYP: Children and young people
HIV: Human immunodeficiency virus
ISARIC: International Severe Acute Respiratory and Emerging Infection Consortium
ICU: Intensive care unit
IQR: Interquartile range
PASC: Post-acute sequelae of SARS-CoV-2 infection
PCC: Post-COVID-19 condition
PCR: Polymerase chain reaction
REDCap: Research Electronic Data Capture
SARS CoV-2: Severe acute respiratory syndrome-related coronavirus 2
STROBE: Strengthening the Reporting of Observational Studies in Epidemiology
WHO: World Health Organization
