# Supplementary material for: Event rates and incidence of post-COVID-19 condition in hospitalised SARS-CoV-2 positive children and young people and controls across different pandemic waves: exposure-stratified prospective cohort study in Moscow (StopCOVID)
Source: BMC Med. 2024 Feb 1;22:48. doi: 10.1186/s12916-023-03221-x (PMC10835884; doi:10.1186/s12916-023-03221-x)

# **Supplementary Materials**

**Event rates and incidence of Post-COVID-19 Condition in Hospitalised SARS-CoV-2 Positive Children and Young People and Controls Across Different Pandemic Waves: Exposure-stratified prospective cohort study in Moscow (StopCOVID)**

# Ekaterina Pazukhina MSc^1,2^*, Mikhail Rumyantsev BSc^3^*, Dina Baimukhambetova BSc^3^*, Elena Bondarenko MD^3^*, Nadezhda Markina MD^3^*, Yasmin El-Taravi MD^3^*, Polina Petrova MD^3^*, Anastasia Ezhova MD^3^*, Margarita Andreeva MD^3^, Ekaterina Iakovleva MD^3^, Polina Bobkova MD^3^, Maria Pikuza MD^3^, Anastasia Trefilova MD^3^, Elina Abdeeva MD^3^, Aysylu Gamirova MD^3^, Yulia Filippova MD^3^, Anastasiia Bairashevskaia BSc^3^, Aleksandr Zolotarev BSc^3^, Nikolay Bulanov MD PhD^4^, Audrey DunnGalvin PhD^3,9^, Anastasia Chernyavskaya MD^10^, Elena Kondrikova MD PhD^3^, Anastasia Kolotilina MD PhD^3^, Svetlana Gadetskaya MD PhD^3^, Yulia V. Ivanova MD PhD^3^, Irina Turina MD PhD^3^, Alina Eremeeva MD PhD^3^, Ludmila A Fedorova MD PhD^3^, Pasquale Comberiati MD^11^, Diego G Peroni MD PhD^11^, Nikita Nekliudov MD^12^, Jon Genuneit MD PhD^13^, Luis Felipe Reyes MD PhD^14,15^, Caroline LH Brackel MD^16,17^, Lyudmila Mazankova MD PhD^20^, Alexandra Miroshina MD PhD^21^, Elmira Samitova MD PhD^20,21^, Svetlana Borzakova MD PhD^8,22^, Gail Carson MD PhD^23^, Louise Sigfrid MD PhD^23^, Janet T Scott MD PhD^24^, Sammie McFarland MSc^25^, Matthew Greenhawt MD MBA^26^, Danilo Buonsenso MD^27,28,29^, Malcolm G Semple MD PhD^30,31^, John O Warner MD FMedSci^32^, Piero Olliaro MD PhD^23^, Ismail M Osmanov MD PhD^8,21^*, Anatoliy A Korsunskiy MD PhD^3^*, Daniel Munblit MD PhD^3,7,33^* and Sechenov StopCOVID Research Team

1. Laboratory of Health Economics, Institute of Applied Economic Studies, The Russian Presidential Academy of National Economy and Public Administration, Moscow, Russia
2. Center for Advanced Financial Planning, Macroeconomic Analysis and Financial Statistics, Financial Research Institute of the Ministry of Finance of the Russian Federation, Moscow, Russia
3. Department of Paediatrics and Paediatric Infectious Diseases, Institute of Child’s Health, Sechenov First Moscow State Medical University (Sechenov University), Moscow, Russia
4. Tareev Clinic of Internal Diseases, Sechenov First Moscow State Medical University (Sechenov University), Moscow, Russia
5. Clinic of Pulmonology, Sechenov First Moscow State Medical University (Sechenov University), Moscow, Russia
6. Department of Internal Medicine №1, Institute of Clinical Medicine, Sechenov First Moscow State Medical University (Sechenov University), Moscow, Russia
7. Research and Clinical Center for Neuropsychiatry, Moscow, Russia
8. Pirogov Russian National Research Medical University, Moscow, Russia
9. School of Applied Psychology, University College Cork, Cork City, Ireland
10. Department of paediatrics and paediatric rheumatology, Sechenov First Moscow State Medical University (Sechenov University), Moscow, Russia
11. Department of Clinical and Experimental Medicine, Section of Pediatrics, University of Pisa, Pisa, Italy
12. Institute for Health Metrics and Evaluation, University of Washington, Seattle, WA, United States
13. Pediatric Epidemiology, Department of Pediatrics, Medical Faculty, Leipzig University, Leipzig, Germany
14. Universidad de La Sabana, Chía, Colombia; Clínica Universidad de La Sabana, Cundinamarca, Colombia
15. Pandemic Sciences Institute, University of Oxford, Oxford, United Kingdom
16. Department of Pediatric Pulmonology, Emma Children's Hospital, Amsterdam University Medical Centers, Amsterdam, the Netherlands
17. Department of Pediatrics, Tergooi MC, Hilversum, the Netherlands
18. Sechenov First Moscow State Medical University (Sechenov University), Moscow, Russia
19. Institute for Regenerative Medicine, Sechenov First Moscow State Medical University (Sechenov University, Moscow, Russia
20. Russian Medical Academy of Continuous Professional Education of the Ministry of Healthcare of the Russian Federation, Moscow, Russia
21. ZA Bashlyaeva Children's Municipal Clinical Hospital, Moscow, Russia
22. Research Institute for Healthcare Organization and Medical Management of Moscow Healthcare Department, Moscow, Russia
23. Nuffield Department of Medicine, ISARIC Global Support Centre, University of Oxford, Oxford, UK
24. MRC-University of Glasgow Centre for Virus Research, Glasgow, UK
25. Long Covid Kids & Friends Charity, Crowhurst, UK.
26. Department of Pediatrics, Section of Allergy/Immunology, Children's Hospital Colorado, University of Colorado School of Medicine, United States
27. Department of Woman and Child Health and Public Health, Fondazione Policlinico Universitario A. Gemelli IRCCS, Rome, Italy
28. Dipartimento di Scienze Biotecnologiche di Base, Cliniche Intensivologiche e Perioperatorie, Università Cattolica del Sacro Cuore, Rome, Italy
29. Center for Global Health Research and Studies, Università Cattolica del Sacro Cuore, Roma, Italia
30. Health Protection Research Unit in Emerging and Zoonotic Infections, Institute of Infection, Veterinary and Ecological Sciences, Faculty of Health and Life Sciences, University of Liverpool, Liverpool, UK
31. Department of Respiratory Medicine, Alder Hey Children's Hospital, Liverpool, UK
32. Inflammation, Repair and Development Section, National Heart and Lung Institute, Faculty of Medicine, Imperial College London, London, United Kingdom
33. Care for Long Term Conditions Division, Florence Nightingale Faculty of Nursing, Midwifery and Palliative Care, King's College London, London, United Kingdom

*Authors contributed equally to the paper.

Table of Contents

Supplementary Materials 1

Sechenov Stop COVID Research Team (Group authors) 4

Table S1. Criteria for hospital admission as per local clinical guidelines. 5

Table S2. Demographic characteristics of study participants – initial and matched cases, sensitivity analysis. 17

Figure S1. Time of study participants hospital admission and their chronological correspondence to COVID-19 variant dominance in Moscow city. 19

Figure S2. Incidence of post‐COVID‐19 condition manifestations in matched exposed and reference groups, sensitivity analysis. 20

Figure S3. Rates of events for incomplete recovery and emotional behavioural changes in matched exposed and reference groups, sensitivity analysis. 21

## Sechenov Stop COVID Research Team (Group authors)

Khazhar Aktulaeva^1^, Islamudin Aldanov^1^, Nikol Alekseeva^1^, Ramina Assanova^1^, Asmik Avagyan^1^, Irina Babkova^1^, Lusine Baziyants^1^, Anna Berbenyuk^1^, Tatiana Bezbabicheva^1^, Julia Chayka^1^, Iuliia Cherdantseva^1^, Yana Chervyakova^1^, Tamara Chitanava^1^, Alexander Chubukov^1^, Natalia Degtiareva^1^, Gleb Demyanov^1^, Semen Demyanov^1^, Salima Deunezhewa^1^, Aleksandr Dubinin^1^, Anastasia Dymchishina^1^, Murad Dzhavadov^1^, Leila Edilgireeva^1^, Yulia Filippova^1^, Veronika Filippova^1^, Yuliia Frumkina^1^, Anastasia Gorina^1^, Cyrill Gorlenko^1^, Marat Gripp^1^, Mariia Grosheva^1^, Eliza Gudratova^1^, Elena Iakimenko^1^, Margarita Kalinina^1^, Ekaterina Kharchenko^1^, Anna Kholstinina^1^, Bogdan Kirillov^1^, Herman Kiseljow^1^, Natalya Kogut^1^, Polina Kondrashova^1^, Irina Konova^2^, Mariia Korgunova^1^, Anastasia Kotelnikova^1^, Alexandra Krupina^1^, Anna Kuznetsova^1^, Anastasia Kuznetsova^1^, Anna S. Kuznetsova^1^, Anastasia Laevskaya^1^, Veronika Laukhina^1^, Baina Lavginova^1^, Yulia Levina^1^, Elza Lidjieva^1^, Anastasia Lindt^1^, Juliya Lyaginskaya^1^, Ekaterina Lyubimova^1^, Shamil Magomedov^1^, Daria Mamchich^1^, Rezeda Minazetdinova^1^, Artemii Mingazov^1^, Anna Mursalova^1^, Daria Nikolaeva^1^, Alexandra Nikolenko^1^, Viacheslav Novikov^1^, Georgiy Novoselov^1^, Ulyana Ovchinnikova^1^, Veronika Palchikova^1^, Kira Papko^1^, Mariia Pavlova^1^, Alexandra Pecherkina^1^, Sofya Permyakova^1^, Erika Porubayeva^1^, Kristina Presnyakova^1^, Maksim Privalov^1^, Alesia Prutkogliadova^1^, Anna Pushkareva^1^, Arina Redya^1^, Anastasia Romanenko^1^, Filipp Roshchin^1^, Diana Salakhova^1^, Maria Sankova^1^, Ilona Sarukhanyan^1^, Viktoriia Savina^1^, Ekaterina Semeniako^1^, Valeriia Seregina^1^, Anna Shapovalova^1^, Khivit Sharbetova^1^, Nataliya Shishkina^1^, Anastasia Shvedova^1^, Valeriia Stener^1^, Valeria Ustyan^1^, Yana Valieva^1^, Maria Varaksina^1^, Katerina Varaksina^1^, Ekaterina Varlamova^1^, Natalia Vlasova^1^, Margarita Yegiyan^1^, Nadezhda Ziskina^1^, Daniella Zolochevskaya^1^, Elena Zuykova^1^

1. Sechenov First Moscow State Medical University (Sechenov University), Moscow, Russia
2. ZA Bashlyaeva Children’s Municipal Clinical Hospital, Moscow, Russia

***The names of the authors are in alphabetic order.***

##

## Table S1. Criteria for hospital admission as per local clinical guidelines.

| **Revision #** | **Release date** | **Case definitions** | **Criteria for hospitalisation** | **Severity** | **Treatment** |
| --- | --- | --- | --- | --- | --- |
| COVID-19 in children Temporary guidelines  Version 1 | 03.04.2020 | **Suspected case**  - presence of clinical manifestations of acute respiratory infection, bronchitis, pneumonia, ARDS, sepsis combined with the following epidemiological history - return from a foreign trip 14 days before the onset of symptoms; - close contacts in the past 14 days with persons under surveillance for SARS-CoV-2 infection who have subsequently become ill; - close contacts in the last 14 days with persons who have a laboratory-confirmed diagnosis of COVID-19.  **Confirmed case** A positive laboratory result for the presence of SARS-CoV-2 RNA by PCR, regardless of clinical manifestations.  **In neonates:**  - at least one clinical symptom, including unstable body temperature, low activity or malnutrition, or shortness of breath;  - changes on a chest X-ray showing abnormalities, including unilateral or bilateral "frosted glass" changes;  - presence of people with confirmed COVID-19 infection among family members or caregivers or  - close contact with people with confirmed COVID-19 infection or patients with severe pneumonia | - children with a severe clinical picture of the disease having a combination of two or more signs against a background of fever: body temperature ≥38.50C, RR ≥30, Sp02 ≤94%  - children with a severe clinical picture of the disease and atypical course of SARS and influenza, community-acquired pneumonia  - children at risk, including those with congenital malformations, chronic diseases of the bronchopulmonary, cardiovascular and endocrine systems, immunodeficiency | **Asymptomatic -** positive test for SARS-CoV-2 RNA, no clinical signs and no visual changes on x-ray (CT)  **Mild -** symptoms of intoxication (fever, fatigue, myalgia) and upper respiratory tract involvement (cough, sore throat, runny nose and sneezing). On examination: changes in the oropharynx; no auscultatory changes in the lungs. In some cases - no fever or only gastrointestinal symptoms (nausea, vomiting, abdominal pain and diarrhoea).  **Mild-to-moderate** - fever, cough (mainly dry unproductive) and pneumonia. Rales (dry or moist) may be heard, but there are no obvious signs of respiratory failure (dyspnoea) and hypoxemia. In some cases - no obvious clinical signs of lower respiratory tract involvement, but a chest CT scan reveals slight changes in the lungs.  **Severe** - early symptoms of acute respiratory infection (fever, cough), which may be accompanied by gastrointestinal symptoms (diarrhoea). The disease usually progresses within a week, with signs of respiratory failure (dyspnoea with central cyanosis) and SpO2 ≤92%. Signs of pneumonia on chest X-ray and CT scan  **Critical** - rapid progression of illness and development of acute respiratory distress syndrome (ARDS) or severe respiratory failure. Shock, encephalopathy, myocardial damage or heart failure, coagulation disorders and acute renal damage, and multiple organ failure may also occur. | **China (Zhejiang University Medical School)**  **Asymptomatic and mild -** Interferon alfa-2b (via nebuliser)  **Moderate and severe** - Interferon alfa-2b (via nebuliser), Lopinavir / Ritonavir  **Critical** - Interferon alfa-2b (via nebuliser), Lopinavir / Ritonavir  **China (Beijing Pediatric Research Institute)**  Interferon alfa-2b (nebulizer or nasal and oropharyngeal spray), Lopinavir/Ritonavir, Umifenovir, Oseltamivir - no recommended algorithm indicated due to insufficient data  **Iran (consensus)**  **Asymptomatic and mild:**  - with risk factors - Oseltamivir + Hydroxychloroquine - without risk factors – Oseltamivir  **Moderate and severe –** Combinations of Oseltamivir + Hydroxychloroquine + Lopinavir/Ritonavir  **Critical** – Combinations of Oseltamivir + Hydroxychloroquine + Lopinavir/Ritonavir (+ Ribavirin)  **United Kingdom (Alder Hey Children's Hospital)**  No recommendations with a comment: there is no evidence that any antiviral treatment or interferon in children is effective.  **USA (University of Michigan Medical School)**  **Moderate, severe, critical -** Remdesivir (specifically approved in the trial) Tocilizumab (as indicated in critical care) |
| The features of the clinical manifestations and treatment of the disease, disease caused by a new coronavirus COVID-19 infection in children. Version 2  **AND**  Temporary guidelines on "Prevention, diagnosis and treatment of new coronavirus infection COVID-19", version 17 | 03.07.2020  14.12.2022 | **Suspected case**  Clinical signs of acute respiratory infection (body temperature > 37.5 °C and one or more signs: cough, dry or with scanty sputum, shortness of breath, feeling of tightness in the chest, SpO2 ≤ 95%, sore throat, nasal congestion, disturbance or loss of sense of smell (hyposmia or anosmia), loss of taste (dysgeusia), weakness, muscle pain, headache, vomiting, diarrhea, skin rash) in absence of other known causes that explain the clinical picture regardless of epidemiological anamnesis.  **Probable (clinically confirmed) case**  1. Clinical manifestations of an acute respiratory infection (t > 37.5 °C and one or more signs: cough, dry or with poor sputum, shortness of breath, chest congestion, SpO2 ≤ 95%, sore throat, nasal congestion, respiratory tract infection. sore throat, stuffy nose, impaired or lost sense of smell (hyposmia or nausea, impaired or lost sense of smell (hypomia or anosmia), loss of taste (dysgeusia), weakness, muscle pain, headache, nausea, vomiting, diarrhoea, headache, eye strain, skin rash in the presence of at least one of the epidemiological signs:  - returning from an overseas trip 14 days before the onset of symptoms; - close contact in the last 14 days with a person under surveillance for SARS-CoV-2 infection, who subsequently became ill; - close contact in the past 14 days with persons who have laboratory-confirmed diagnosis of COVID-19.  2. Presence of the clinical manifestations described in p.1, in combination with characteristic CT abnormalities in the lungs, regardless of a single laboratory test for SARS-CoV-2 RNA and epidemiological history.  3. Presence of clinical signs (listed in p.1), combined with characteristic changes in the lungs on radiological examination when laboratory examination for the presence of SARS-CoV-2 RNA is impossible  **Confirmed case**  A positive laboratory result for the presence of SARS-CoV-2 RNA by PCR regardless of clinical manifestations | **1. Fever above 38.5 °C** on the day of admission or fever above 38.0 °C for 5 days or more.  **2. Respiratory distress (presence of any of the following respiratory distress symptoms):** - Tachypnea: respiratory rate in children under 1 year of age more than 50, between 1 and 5 years of age more than 40, over 5 years of age more than 30 per min  - Dyspnoea at rest or when the child is restless  - Participation of accessory muscles in the act of breathing  - Retractions of the chest wall when breathing  - Flaring of the wings of the nose when breathing  - Wheezing or moaning breathing  - Episodes of apnoea  - Nodding head movements, synchronised with inhalation  - Distant wheezing  - Inability to suck/drink due to respiratory disturbances  - Acrocyanosis or central cyanosis  - Blood oxygen saturation on pulse oximetry SpO2 < 95%  **3. Tachycardia in children** under 1 year old over 140, between 1 and 5 years old over 130, over 5 years old over 120 per min.  **4. Presence of haemorrhagic rash**  **5. Presence of any of the emergency and urgent signs:** - Convulsions  - Shock  - Severe respiratory distress  - Severe dehydration  - Decreased consciousness (drowsiness) or agitation  **6. Presence of severe background illness regardless of fever and respiratory depression:** - Immunodeficiency status, including treatment with immunosuppressive drugs  - Oncological and oncohematological diseases - Diseases with disorders of the blood-clotting system  - Congenital and acquired heart defects and diseases, including rhythm disorders, cardiomyopathy  - Congenital and acquired chronic lung diseases  - Diseases of the endocrine system (diabetes mellitus, obesity)  - Chronic serious liver, kidney and gastrointestinal diseases  **7. Failure to isolate when living with people from risk groups**  **8. Lack of conditions for treatment at home or guarantees of recommendations** (dormitories, social welfare institutions, emergency accommodation, socially disadvantaged family, adverse social and domestic conditions). | **Asymptomatic -** positive results of laboratory testing for SARS-CoV-2 RNA, no clinical signs of the disease and no visual clinical signs of the disease; no visual changes on the radiograph.  **Mild** - fever < 38.5 °C, symptoms of intoxication (weakness, myalgia) and upper respiratory tract involvement (cough, sore throat, nasal congestion). upper respiratory tract (cough, sore throat, stuffy nose). - on examination: changes in the oropharynx; no auscultatory changes in the lungs. - in some cases - no fever or only Gastrointestinal symptoms (nausea, vomiting, stomach pain and diarrhoea) or skin rashes only.  **Mild-to-moderate** - fever > 38.5 °C, cough (mostly dry unproductive), pneumonia. - rales (dry or moist) may be heard, but there are no obvious signs of respiratory failure (dyspnoea) and hypoxemia, SpO2 is > 93%. - in some cases there may be no obvious clinical signs of lower respiratory tract involvement but a chest computed tomography (CT) scan reveals slight changes in the lungs. chest CT scan demonstrates small changes in the lungs typical of mild to moderate levels of viral lung injury. mild to moderate viral lung disease (CT1-2)  **Severe** - symptoms of acute respiratory infection at the beginning of the illness (fever, cough), which may be accompanied by gastrointestinal symptoms (diarrhoea). The disease usually progresses within a week,  - signs of respiratory failure appear (dyspnoea with central cyanosis), SpO2 is 93%. - signs of pneumonia on chest X-ray and CT scan typical of severe or critical viral lung disease (CT3-4)  **Critical form - multisystem inflammatory syndrome (cytokine storm, Kawasaki-like syndrome)**  - fever > 24 h,  - multisystem (> 2) involvement of different organs (heart, kidney and central nervous system involvement, respiratory symptoms, possible development of ARDS, gastrointestinal symptoms, haematological disorders, skin rash, myalgia, arthralgia). - various laboratory markers are elevated: neutrophilic leukocytosis with lymphopenia, increased levels of C-reactive protein (CRP), procalcitonin, sed rate, LDH, transaminases, triglycerides, interleukin 6, ferritin and D-dimer, hypoalbuminemia. Hypercoagulation, DIC, thrombosis and thromboembolic complications are possible. - warm vasoplegic shock may develop, refractory to correction of circulating blood volume, requiring administration of norepinephrine.  - at cardiac ultrasound - decreased ejection fraction, coronary artery disease and sometimes coronary artery aneurysms  - in the development of macrophage activation syndrome (haemophagocytic syndrome, HFS): febrile fever, refractory to antimicrobial therapy, ARDS, lymphadenopathy, multiple organ failure.  - in laboratory tests: significant increases in CRP, ferritin, LDH, AST, ALT, serum triglycerides, hyponatremia, hyperbilirubinemia, increased serum procalcitonin, elevated levels of D dimer, blood fibrin degradation products, hypofibrinogenemia, rapid decrease in CRP and leucocyte count, despite high inflammatory activity, thrombocytopenia, two- or three-stage cytopenia, consumption coagulopathy | **Asymptomatic -** no etiotropic therapy is required.  **Mild (acute respiratory infections, non-serious pneumonia)**  1. Symptomatic treatment. 2. Prescription of antivirals may be considered in children at risk who have severe comorbidities, immunodeficiency (by decision of the medical committee)  **Moderate (pneumonia with respiratory failure)**  **Oxygen therapy:** up to 2 months. - 0.5-1 l/min, 2 months to 5 years 1-2 l/min, > 5 years 2-4 l/min.  The prescription of antivirals may be considered in children at risk who have severe comorbidities or immunodeficiency (by decision of the medical committee).  **1. Anticoagulants:** low molecular weight heparins at a prophylactic dose under coagulogram monitoring  **2. Symptomatic treatmen**t  **3. Antibiotic therapy:** - is not indicated in the absence of signs of bacterial inflammation or progression to severe!  - Amoxicillin is indicated for suspected community-acquired (pneumococcal) pneumonia; if there are risk factors for drug resistance (hospitalization or antibiotic treatment in the previous 3 months, attendance at a day-care centre or sibs, chronic illness) - amoxicillin/clavulanate at 90 mg/kg/day or ceftriaxone 80 mg/kg/day is indicated.  - If atypical community-acquired pneumonia (mycoplasma) is suspected, a macrolide is prescribed.  - For nosocomial pneumonia/bacterial superinfection, choose an antimicrobial based on microbiological findings and, if no culture is possible, empirically based on previously used antimicrobials.  **Severe**  1. Oxygen therapy: up to 2 months. - 0.5-1 l/min, 2 months to 5 years - 1-2 l/min, >5 years - 2-4 l/min. 2. Anticoagulants: low-molecular-weight heparins in a therapeutic dose, in heparin-induced thrombocytopenia (HIT) - fondaparinux, rivaroxaban under coagulogram and thromboelastogram control. 3. Dexamethasone 10 mg/m2 body surface/injection or methylprednisolone 0.5-1 mg/kg/injection intravenously every 12 hours (see below). 4. Human normal immunoglobulin at a dose of 0.3-0.4 g/kg. 5. Antibiotic therapy is indicated if a secondary bacterial infection is suspected. The choice of antimicrobials is based on the extent and severity of the secondary bacterial infection. 6. Symptomatic treatment.  In children aged ≥15 years may be considered the etiotropic prescribing tactics, recommended for adults  **Multisystem inflammatory syndrome**  1. Oxygen therapy. 2. Anticoagulants: low molecular weight heparins, in HIT: fondaparinux, rivaroxaban under coagulogram and thromboelastogram control. 3. Dexamethasone 10 mg/m2 body surface/day intravenously or methylprednisolone 0.5-1 mg/kg/day/intravenous infusion every 12 hours. 4 Tocilizumab 4-8 mg/kg intravenously once (if HC is ineffective and there are no contraindications). 5. If GC + tocilizumab are ineffective - canakinumab 4-8 mg/kg/injection intravenously once (if there are no contraindications). 6. Human normal immunoglobulin at a dose of up to 2 g/kg. 7. Acetylsalicylic acid 50-100 mg/kg orally (for coronary artery disease/expansion/aneurysis). 8. Antibiotic therapy is indicated if secondary bacterial infection is suspected. The choice of antimicrobials is based on the extent and severity of the secondary bacterial infection. 9. Symptomatic treatment 10. Cardiotonics and vasopressors for the development of shock |
| Clinical protocol for the treatment of children with a new coronavirus infection (COVID-19), in inpatient care in medical organizations of the state health care system of the city of Moscow of the Moscow health care system | 2021 | **Suspected case** - clinical signs of severe pneumonia, ARDS, sepsis  AND epidemiological criteria - travel from abroad in the past 14 days OR - direct contact with people currently under surveillance for suspected 2019-nCoV that had become symptomatic later OR - direct contact with people with 2019-nCoV confirmed by testing)  **Probable case** – Unspecified  **Confirmed case** Positive laboratory test results on PCR RNA 2019-nCoV independent from clinical symptoms | 1. Severe to moderate respiratory illness or community-acquired pneumonia.  2. Fever > 38.5 °C, including history of fever, or fever < 36.0 °C, or a fever > 38.0 °C for more than five days.  3. Dyspnoea at rest or with restlessness, or non-fever-related tachypnoea, > 15% of age-standard (under 1 year - > 50 BPM, 1 to 5 years old - > 40 BPM, over 5 years old - > 30 BPM).  4. Non-fever-related tachycardia > 15 % of age-standard (under 1 year old > 140 BPM, 1 to 5 years - > 130 BPM, over 5 years of age - > 120 BPM).  5. SpO2 ≤ 95 %.  6. Decreased consciousness (drowsiness) or increased agitation, inversion of sleep, refusal to eat or drink.  7. Seizures  8. No positive changes or an increase in clinical symptoms after 5 days after the onset of the disease  9. Failure to isolate when living with people from risk groups  10. Lack of conditions for home treatment or guarantees of compliance with recommendations (dormitories, social welfare institutions, reception centre, socially disadvantaged family, poor social and living conditions).  10. Children in the first year of life  11. Children with current or past COVID-19 or who have been in contact with COVID-19 patients, if fever > 38°C is combined with conjunctivitis, cheilitis, swelling of the palms and/or feet, rash of any nature, enlarged lymph nodes (in order to exclude Kawasaki disease and MIS).  12. The presence of severe underlying diseases:  - **cardiology** ( Q20-28, I 20–I28, I30, I33, I38, I40, I41, I42, I43, I45.6, I47, I48, I49.8 (Brugada syndrome, long QT interval syndrome), I50  - **rheumatology** (M08.2 (systemic JIA); M30.0; M30.1; M30.2-8; (vasculitis); M31.0; 31.3; M31.4; M31.7 (vasculitis), M32.0; M32.1; M32.8; M32.9 (vasculitis), M33.0; M33.1; M33.2; M33.9 (juvenile dermatomyositis), M34.0-9 (systemic scleroderma), M35.0-9 (Sjögren's, Behçet's, etc.))  - **nephrology** (N03.0; N04; N11-N16; N18.0-9; N19)  - **haematology** (C81; C82-C86; C96; C88; C90; C91.0-9; C92.0-9; C93.09; C94.0,2,3,4,6,7; C95.0; C94.3,6,7; C95.1-9; D45-D47; D55-89)  - **immunology** (D80; D80.0; D80.1; D80.3; D80.5; D80.6; D80.8; D80.9; D81; D82; D83; D84; D89; G11.3; Е31.0)  - **pulmonology** (J41.8, J45; J82; J84; E84)  **- endocrinology** (E10.2-9; E11.2-9; Е66-68; Q87.1; Q87.8; E24.0; E24.2; E24.8; E24.9; Е27.0)  - **oncology** (C00 – С97) | **Asymptomatic -** no clinical signs of the disease and no changes in the lung X-ray/CT. The results of the laboratory SARS-CoV-2 RNA positive  **Mild -** fever < 38.0 °C, symptoms of intoxication (weakness, myalgia) and upper respiratory tract involvement (cough, sore throat, stuffy nose).  - on examination: changes in the oropharynx; no auscultatory changes in the lungs.  - in some cases - gastrointestinal symptoms only (nausea, vomiting, abdominal pain and diarrhoea) or abdominal pain and diarrhoea) or only skin rashes. SpO2 > 95%  **Moderate -** fever > 38.0 °C, cough (mainly dry non-productive).  - Rales (dry or moist) may be heard, but there are no obvious signs of respiratory distress (dyspnea) and hypoxemia, SpO2 is > 93%.  - Clinical symptoms of lower respiratory tract involvement  may not be present, but a computer tomography (CT) scan of the lungs findings are typical of viral lung injury of mild to moderate severity (CT1-2).  **Severe -** Symptoms of acute respiratory infection at the beginning of the illness (fever, cough), which may be accompanied by gastrointestinal symptoms (diarrhoea). The disease usually progresses within a week, with signs of respiratory failure (dyspnoea with central cyanosis), SpO2 ≤ 93%.  - Signs of lung involvement on chest X-ray and CT scan signs of lung damage on chest X-ray and chest CT, typical for viral interstitial severe or critical lung lesions (CT3-4).  **Extreme severe -** acute respiratory distress syndrome (ARDS).  - Multisystemic inflammatory syndrome (MIS):  - MIS with signs of acute COVID-19;  - MIS developing after 3-4 weeks (PCR "+");  - MIS developing 3-4 weeks after an acute COVID-19, with signs of Kawasaki syndrome (PCR "-");  - MIS with myocarditis, shock, multiple organ failure and high ferritin (at any stage of the disease).  - fever > 24 h, multisystem (> 2) involvement of different organs (heart, kidneys, central nervous system involvement, respiratory symptoms, possible development of ARDS, gastrointestinal symptoms, hematological disorders, skin rashes, myalgia, arthralgia).  - There is an increase in various laboratory markers: neutrophilic leukocytosis with lymphopenia, increased levels of CRP, procalcitonin, sedimentation, LDH, transaminases, troponin, natriuretic peptide (NTproDNP), triglycerides, interleukin-6, ferritin and D-dimer, hypoalbuminemia.  - Hypercoagulability, DIC, possible thrombosis and thromboembolic complications.  - Possible development of warm vasoplegic shock, refractory to the correction of circulating blood volume, requiring norepinephrine injection. norepinephrine.  - Cardiac ultrasound reveals left ventricular cavity dilatation and reduced ejection fraction, mitral valve insufficiency, coronary artery dilatation, thickening of the coronary artery walls and in some cases - formation of coronary aneurysms.  - Haemophagocytic syndrome (HPS): febrile fever, refractory to antimicrobial therapy, ARDS, lymphadenopathy, multiple organ failure. In laboratory tests: significant increase in CRP, ferritin, LDH, AST, ALT, serum triglycerides, hyponatremia, hyperbilirubinemia, increased serum procalcitonin, increased D-dimer, fibrin degradation products, hypofibrinogenemia, rapid decrease of CRP and leucocyte count, despite high inflammatory activity, thrombocytopenia, two- or three-stage cytopenia, consumption coagulopathy. | **Asymptomatic**  **In non-at-risk children** - no etiotropic therapy is indicated.  **For children from risk groups** - monoclonal antibody-based therapies until day 10 of onset of symptoms or when PCR is positive for SARS-CoV-2 RNA (Sotrovimab, Bamlanivimab+ Etsevimab, Casirivimab+Imdevimab).  **Mild**  **3-14 days:**   - Mandatory - Recombinant IFN-alpha-2b (intranasal, gel or per rectum) or Umifenovir. - In children from risk groups - up to day 10 from the appearance of the first of symptoms or PCR "+", "-", or a positive test for SARS-CoV-2 antigen: Sotrovimab, Bamlanivimab+ Etsevimab, Casirivimab+Imdevimab - Etiotropic therapy   **15 days and more:** symptomatic therapy  **Moderate**  **3-10 days:**   - Mandatory - Recombinant IFN-alpha 2b (intranasal, gel or per rectum) Remdesivir. - In children from risk groups – up to day 10 from the appearance of the first symptoms or PCR "+", "-", or a positive test for SARS-CoV-2 antigen: Sotrovimab, Bamlanivimab+etsevimab, Casirivimab+Imdevimab. - Immunoglobulin against COVID-19 (Covid globulin) - Symptomatic therapy - By indication - antibiotics (in the presence of bacterial complications, elevated markers inflammatory markers - leukocytosis, increased sedimentation, increased CRP, increased procalcitonin) - Anticoagulant therapy: low molecular weight heparins under coagulogram control   **10-14 days:**   - Mandatory - recombinant IFN-alpha-2b (intranasal, gel or per rectum) - Symptomatic therapy - By indication:   - antibiotics (in the presence of bacterial complications, increased inflammatory markers - leukocytosis, increased CRP, increased CRP, increased procalcitonin)  - anticoagulant therapy: low molecular weight heparins under monitored coagulogram  **15 days and more:**   - Symptomatic therapy - By indication - antibiotics (in the presence of bacterial complications, elevated markers inflammatory markers - leukocytosis, increased sedimentation, increased CRP, increased procalcitonin) - Anticoagulant therapy: low molecular weight heparins under coagulogram control   **Severe**  **Mandatory:**  - Anticoagulant therapy: low molecular weight heparins at therapeutic dose, in case of heparin-induced thrombocytopenia (HIT): fondaparinux, rivaroxaban under coagulogram (including D-dimer) and thromboelastogram monitoring.  - Glucocorticosteroids: methylprednisolone  - Antibacterial therapy  **By indication:** Remdesivir  **In children from risk groups** – up to day 10 from the first symptoms, either PCR '+', '-' or a positive SARS-CoV-2 antigen test: Sotrovimab, Bamlanivimab+ Etsevimab, Casirivimab+Imdevimab  Human immunoglobulin normal;  Human immunoglobulin against COVID-19 (Covid-Globulin);  **Antiviral plasma in the absence of ARDS**  - Interleukin 6 receptor inhibitor: tocilizumab  or  - Interleukin 1 inhibitors - canakinumab or anakinra  **Symptomatic therapy**  **Critical (extreme severe)**  **Mandatory:**  - Antibacterial therapy;  - Anticoagulant therapy: low-molecular-weight heparins, in case of heparin-induced thrombocytopenia (HIT) fondaparinux, rivaroxaban under coagulogram and thromboelastogram monitoring.  - Glucocorticosteroids: dexamethasone or methylprednisolone  **By indication:**  - Remdesivir  - Sotrovimab, Bamlanivimab+ Etesevimab, or Casirivimab+Imdevimab  - Human immunoglobulin normal  **Antiviral plasma in the absence of ARDS**  - Interleukin 6 receptor inhibitor: tocilizumab  or  - Interleukin 1 inhibitors - canakinumab or anakinra  - Symptomatic therapy |
| Acute respiratory viral infection in children. Clinical guidelines of The Union of Pediatricians of Russia | 13.09.2022 | **Suspected case**  - nasal congestion and nasal discharge (clear and/or white and/or yellow and/or green)  - fever (usually lasts no more than 3 days and then go down. In some infections (influenza and adenovirus), a fever of more than 38ºC may last for a longer period of time (up to 5-7 days)  - sore throat, coughing, red eyes and sneezing  - direct contact with people currently under surveillance for suspected ARVI | - children under 3 months of age with febrile fever due to their high risk of developing a severe bacterial infection;  - children of any age with any of the following symptoms (main danger signs): inability to drink / breastfeed; drowsiness or unconsciousness; respiratory rate less than 30 per minute or apnoea; symptoms of respiratory distress; central cyanosis; heart failure phenomena; severe dehydration;  - children with complex febrile seizures (lasting more than 15 minutes and/or recurring more than once within 24 hours) are hospitalised for the duration of the fever;  -children with fever and suspected severe bacterial infection (BUT there may be hypothermia!) with the following accompanying symptoms: drowsiness, lethargy; refusal to eat or drink; haemorrhagic skin rash; vomiting;  - children with respiratory distress with any of the following symptoms: wheezing, bloating of the wings of the nose when breathing, nodding movements (head movements synchronised with breathing in); respiratory rate at 2 months > 60 per minute, 2-11 months > 50 per minute, over 1 year old > 40 per minute; lower chest tightness when breathing; blood oxygen saturation < 92% when breathing with room air | The division of acute respiratory infections (nasopharyngitis, pharyngitis, laryngotracheitis without laryngeal stenosis) by severity is not appropriate. | **Etiotropic therapy** is recommended for influenza A (including H1N1) and B during the first 24-48 hours of illness. Neuraminidase inhibitors may be given: Oseltamivir from 1 year of age at ≤ 15 kg - 30 mg (2 ml) 2 times daily, > 15-23 kg - 45 mg (3 ml) 2 times daily, > 23-40 kg - 60 mg (4 ml) 2 times daily, > 40 kg - 75 mg (5 ml), for 5 days, or Zanamivir in children from 5 years old 2 inhalations (10 mg total) 2 times daily, 5 days.  It has been recommended that **therapeutic topical forms of interferon-alpha** should be considered no later than day 1-2 of illness, but there is no reliable evidence of antiviral efficacy or safety in children.  The use of **systemic antimicrobials is not recommended** for the treatment of uncomplicated acute respiratory infections and influenza, including when the illness is accompanied in the first 10-14 days by rhinosinusitis, conjunctivitis, laryngitis, croup, bronchitis, bronchoobstructive syndrome  **Increased fluid intake** during an acute respiratory infection is not recommended. The benefits of abundant drinking are questionable and increased secretion of antidiuretic hormone during illness, on the contrary, promotes fluid retention.  An elimination therapy is recommended, as this therapy is effective and safe. Injection of sodium chloride (0.9%) or a sterile seawater solution into the nose several times a day removes mucus and restores the functioning of the atrial fibrillation epithelium.  A short course of no more than 5 days of local vasoconstrictors (decongestants and other topical preparations) is recommended. These drugs do not shorten the duration of the runny nose, but may relieve the symptoms of nasal congestion and restore auditory tube function. In children 0-6 years of age, phenylephrine 0.125%, oxymetazoline 0.01-0.025%, xylometazoline 0.05% (from 2 years) are used; the more concentrated solutions are used for older children.  To lower the body temperature of a feverish child, it is advisable to uncover and wipe the child with 25-30°C water.  Only two drugs - paracetamol up to 60 mg/kg/day or ibuprofen up to 30 mg/kg/day - are recommended for reducing body temperature in children due to proven safety.  It is recommended to consider giving a warm drink or, after 4 years of age, using lollipops or lozenges containing antiseptic medication to relieve coughing in pharyngitis, which is associated with a "scratchy throat" due to inflammation of the pharyngeal mucosa or drying out when breathing through the mouth. |
| Influenza in children. Clinical guidelines by The Ministry of Healthcare of Russia | 2017 | **Suspected case**  - chills, headache, aching muscles, joints, pain when the eyeballs are moved or pressed, photophobia, lacrimation, sudden weakness and fatigue, lethargy. Dizziness, fainting, loss of consciousness, delirium, hallucinations and seizures may occur  - direct contact with people currently under surveillance for suspected influenza that had become symptomatic later  - direct contact with people with influenza confirmed by testing)  **Probable case** – Unspecified  **Confirmed case** Positive laboratory test results on PCR RNA Influenza | - severity of the general intoxication syndrome (severe agitation with convulsions and loss of consciousness against a background of hyperthermia);  - haemorrhagic syndrome (nosebleed, blood in sputum, vomit and stool, haemorrhagic rash), development of DIC or Gasser syndrome with formation of AKI;  - haemodynamic instability with the development of centralised peripheral circulation (cold extremities, cyanosis, marbling of the skin against a background of general pallor and hyperthermia, development of collapse and coma);  - severe respiratory failure with diffuse cyanosis and pallor, as well as symptoms of pneumonia and signs of possible ARDS: cough with foaming sputum mixed with blood, shortening of the pulmonary sound on percussion, large number of differently coloured wet rales and abundant crepitation on auscultation, sharp drop in BP, muffled cardiac tones and arrhythmia;  - newborns and children in the first years of life with moderate disease, but with a complicated course and exacerbation of comorbidities | **Mild** - body temperature does not rise above 38.5 °C (may even remain normal), intoxication (chills, malaise, sweating, decreased appetite) and catarrhal syndrome (acute rhinitis, pharyngitis, tracheitis) are mild to moderate. Haemorrhagic manifestations are absent.  **Moderate** - dominant in 86-90% of hospitalised patients, is characterised by a body temperature between 38.5-39.5 °C, and a moderate to severe intoxication syndrome (chills, weakness, headache, dizziness, myalgia, arthralgia, drowsiness or increased excitability, adynamic, vomiting, nausea). In some cases an increase in body temperature, mainly in the form of hyperthermia, may be the only manifestation of the disease. Respiratory tract syndrome is moderate, with the development of rhinitis, pharyngitis, tracheitis and possibly bronchitis. Haemorrhagic syndrome is manifested by nosebleeds, petechial haemorrhagic rash on the skin and mucous membranes, which is moderate and of short duration.  **Severe** - a rise in body temperature of 39.5°C, a pronounced intoxication syndrome with encephalic or meningoencephalic reactions (delirium, hallucinations, seizures, confusion or loss of consciousness, vomiting), haemorrhagic syndrome (nasal bleeding, fine-point or petechial rash on the face, neck, chest and upper extremities, haemorrhages into the mucous membrane of the mouth and nose, back of the throat, conjunctivae, blood in vomit, hemoptysis, haematuria, etc.). Disseminated intravascular coagulation (DIC) syndrome is not uncommon.  **Extreme severe** - haemophagocytic syndrome | It is recommended to use **oseltamivir, zanamivir** as first-line drugs to inactivate the influenza virus, preventing entry and subsequent reproduction of the virus in cells of the respiratory tract. |

## Table S2. Demographic characteristics of study participants – initial and matched cases, sensitivity analysis.

| Characteristic | Initial sample | | | Matched  Wuhan - Reference | | Matched  Omicron - Reference | | Matched  Wuhan - Omicron | |
| --- | --- | --- | --- | --- | --- | --- | --- | --- | --- |
|  | Wuhan variant cohort | Omicron variant cohort | Reference cohort | Wuhan variant cohort | Reference cohort | Omicron variant cohort | Reference cohort | Wuhan variant cohort | Omicron variant cohort |
| Number of participants | 453 | 357 | 376 | 213 | 213 | 273 | 273 | 194 | 194 |
| Median time from the hospital discharge to the follow‐up point (IQR), months | 7.5 (7.3,7.7) / 453 | 6.2 (6.1,6.3) / 357 | 6.2 (6.1,6.4) / 376 | 7.5 (7.3 - 7.7) | 6.2 (6.1 - 6.4) | 6.1 (6.1 - 6.3) | 6.2 (6.1 - 6.3) | 7.5 (7.3 - 7.7) | 6.2 (6.1 - 6.3) |
| Length of hospitalisation, days | 7.0 (4.0,10.0) / 453 | 3.3 (1.8,5.5) / 357 | 3.2 (2.0,5.0) / 375 | 5.0 (3.0 – 7.0) | 4.0 (2.6 - 5.9) | 2.9 (1.72 - 4.94) | 3.0 (1.9 - 4.7) | 4.0 (3.0 – 7.0) | 4.3 (2.2 - 6.0) |
| Gender (female) | 239 / 453 (53%) | 164 / 357 (46%) | 190 / 376 (51%) | 48.6% | 48.6% | 51.1% | 51.1% | 52.3% | 52.3% |
| Median age (IQR) at hospital admission, years | 10.3 (2.8,14.7) / 453 | 2.6 (0.8,7.0) / 357 | 4.2 (1.9,7.8) / 376 | 4.6 (1.5 - 9.9) | 4.5 (1.5 - 10.2) | 3.2 (1.1 - 7.0) | 3.0 (1.4 - 7.1) | 3.2 (1.0 - 10.0) | 3.2 (0.9 - 9.6) |
| Severe COVID‐19 (requiring non‐invasive ventilation or invasive ventilation or ICU) | 11 / 453 (2.4%) | 22 / 357 (6.2%) | 11 / 376 (2.9%) | 0.0% | 0.0% | 2.2% | 2.2% | 0.0% | 0.0% |
| Comorbidities | | | | | | | | | |
| Neurological | 29 / 270 (10.7%) | 13 / 277 (4.7%) | 20 / 375 (5.3%) | 10.7% | 4.7% | 9.3% | 5.5% | 9.7% | 11.5% |
| Neurodisability | 9 / 269 (3.6%) | 2 / 273 (0.7%) | 6 / 373 (1.6%) | 2.4% | 0.5% | 3.7% | 1.5% | 2.7% | 5.3% |
| Heart diseases | 11 / 270 (4.1%) | 12 / 276 (4.4%) | 16 / 373 (4.3%) | 3.4% | 4.2% | 3.8% | 4.0% | 3.8% | 4.3% |
| Respiratory diseases (not including asthma) | 9 / 270 (3.3%) | 3 / 272 (1.1%) | 5 / 372 (1.3%) | 3.9% | 1.0% | 2.6% | 1.1% | 2.2% | 4.1% |
| Tuberculosis | 0 / 211 (0.0%) | 0 / 277 (0.0%) | 0 / 373 (0.0%) | 0.0% | 0.0% | 0.0% | 0.0% | 0.0% | 0.0% |
| Asthma (physician diagnosed) | 3 / 271 (1.1%) | 7 / 277 (2.5%) | 8 / 372 (2.2%) | 1.5% | 2.8% | 0.4% | 2.2% | 1.1% | 0.5% |
| Allergic rhinitis/hay fever | 21 / 267 (7.9%) | 18 / 276 (6.5%) | 25 / 373 (6.7%) | 9.9% | 7.5% | 6.6% | 5.9% | 9.0% | 6.8% |
| Food allergy | 43 / 264 (16.3%) | 31 / 276 (11.2%) | 43 / 372 (12.0%) | 16.8% | 11.9% | 7.8% | 11.3% | 17.1% | 7.9% |
| Atopic dermatitis/Eczema | 27 / 264 (10.2%) | 31 / 274 (11.3%) | 42 / 374 (11.0%) | 10.8% | 12.5% | 1.5% | 11.2% | 11.2% | 1.6% |
| Dermatological problems | 8 / 263 (3.0%) | 5 / 277 (1.8%) | 6 / 372 (1.6%) | 2.9% | 2.4% | 0.7% | 1.8% | 2.2% | 1.1% |
| Intestinal (gut) problems | 30 / 270 (11.1%) | 23 / 279 (8.2%) | 27 / 373 (7.2%) | 11.6% | 9.1% | 7.3% | 6.6% | 13.3% | 8.9% |
| Haematological conditions | 2 / 270 (0.7%) | 3 / 274 (1.1%) | 4 / 373 (1.1%) | 1.4% | 1.4% | 2.2% | 1.1% | 1.6% | 2.1% |
| Malignancy | 0 / 270 (0.0%) | 1 / 276 (0.4%) | 1 / 374 (0.3%) | 0.0% | 0.0% | 0.7% | 0.0% | 0.0% | 0.5% |
| Immunity problems | 2 / 268 (0.8%) | 1 / 277 (0.4%) | 1 / 374 (0.3%) | 0.5% | 0.0% | 1.8% | 0.4% | 0.5% | 2.1% |
| Genetic | 8 / 270 (3.0%) | 1 / 272 (0.4%) | 3 / 373 (0.8%) | 2.5% | 0.0% | 2.2% | 0.4% | 2.1% | 3.2% |
| Diabetes Mellitus | 0 / 271 (0.0%) | 0 / 275 (0.0%) | 0 / 372 (0.0%) | 0.0% | 0.0% | 0.7% | 0.0% | 0.0% | 0.5% |
| Endocrinological conditions | 5 / 264 (1.9%) | 2 / 276 (0.7%) | 2 / 374 (0.5%) | 2.9% | 0.9% | 0.4% | 0.4% | 3.8% | 0.5% |
| Kidney disease | 14 / 267 (5.2%) | 11 / 278 (4.0%) | 14 / 374 (3.7%) | 5.8% | 5.3% | 3.0% | 3.7% | 7.0% | 4.2% |
| Overweight and obesity (as defined by clinical staff) | 5 / 270 (1.9%) | 6 / 275 (2.2%) | 7 / 374 (1.9%) | 1.4% | 2.8% | 0.4% | 1.8% | 1.6% | 0.5% |
| Malnutrition | 3 / 265 (1.1%) | 15 / 276 (5.4%) | 20 / 374 (5.3%) | 1.0% | 5.6% | 2.6% | 5.5% | 0.5% | 2.1% |
| Rheumatologic disorder | 5 / 267 (1.9%) | 1 / 279 (0.4%) | 1 / 374 (0.3%) | 2.4% | 0.0% | 0.0% | 0.4% | 1.6% | 0.5% |
| HIV | 0 / 270 (0.0%) | 0 / 275 (0.0%) | 0 / 371 (0.0%) | 0.0% | 0.0% | 0.0% | 0.0% | 0.0% | 0.0% |
| *^1^* Median (25%,75%) / N; n (%); n / N (%) | | | | | | | | | |

n (%) or median (IQR) excluding missing values. ICU, intensive care unit.

## Table S3. Incidence and prevalence of Post COVID-19 manifestations.

| Outcome | Wuhan variant cohort - Omicron cohort | | | Omicron variant cohort – Reference cohort | | | Wuhan variant cohort - Reference cohort | | |
| --- | --- | --- | --- | --- | --- | --- | --- | --- | --- |
|  | Omicron variant cohort | Wuhan variant cohort | P-value | Omicron variant cohort | Reference  cohort | P-value | Wuhan variant cohort | Reference cohort | P-value |
| **Incidence of Post COVID-19 condition (# events per 1,000 person-months at risk)** | | | | | | | | | |
| At least one symptom | 11 / 762, 15.7 per 1,000 PM (6.5 - 26.7) | 57 / 608, 87.0 per 1,000 PM (61.0 - 118.9) | <0.01** | 15 / 876, 14.1 per 1,000 PM (6.8 - 23.7) | 9 / 868, 10.1 per 1,000 PM (3.4 - 18.2) | 0.45 | 60 / 663, 89.6 per 1,000 PM (64.4 - 120.4) | 8 / 823, 11.0 per 1,000 PM (4.8 - 19.7) | <0.01** |
| **Recovery (event rate, %)** | | | | | | | | | |
| Incomplete recovery | 17 / 243, 7.0% (3.8% - 10.7%) | 33 / 249, 13.3% (8.8% - 18.2%) | 0.03* | 19 / 286, 6.6% (3.9% - 9.9%) | 10 / 288, 3.5% (1.4% - 6.1%) | 0.12 | 37 / 266, 13.9% (9.6% - 18.7%) | 10 / 275, 3.6% (1.4% - 6.3%) | <0.01** |
| **Incidence of Post COVID-19 Manifestations (# events per 1,000 person-months at risk)** | | | | | | | | | |
| Cardiovascular | 0 / 783, 0.0 per 1,000 PM (0.0 - 0.0) | 8 / 681, 4.2 per 1,000 PM (0.0 - 11.6) | 0.22 | 0 / 900, 0.0 per 1,000 PM (0.0 - 0.0) | 1 / 903, 1.1 per 1,000 PM (0.0 - 3.4) | 1 | 7 / 819, 3.6 per 1,000 PM (0.0 - 9.8) | 2 / 839, 1.2 per 1,000 PM (0.0 - 3.6) | 0.62 |
| Dermatological | 0 / 786, 0.0 per 1,000 PM (0.0 - 3.8) | 20 / 729, 21.9 per 1,000 PM (10.7 - 35.8) | <0.01** | 0 / 900, 0.0 per 1,000 PM (0.0 - 3.3) | 1 / 882, 2.2 per 1,000 PM (0.0 - 6.8) | 0.5 | 17 / 792, 21.5 per 1,000 PM (11.2 - 34.4) | 3 / 843, 3.6 per 1,000 PM (0.0 - 8.4) | <0.01** |
| Fatigue | 1 / 781, 2.5 per 1,000 PM (0.0 - 6.5) | 16 / 723, 28.9 per 1,000 PM (16.1 - 45.3) | <0.01** | 2 / 895, 2.2 per 1,000 PM (0.0 - 5.7) | 1 / 894, 1.1 per 1,000 PM (0.0 - 4.5) | 1 | 19 / 786, 31.4 per 1,000 PM (18.7 - 48.0) | 1 / 837, 2.4 per 1,000 PM (0.0 - 6.0) | <0.01** |
| Gastrointestinal | 4 / 788, 5.1 per 1,000 PM (1.3 - 11.9) | 13 / 747, 19.0 per 1,000 PM (9.3 - 31.8) | 0.02* | 7 / 877, 5.7 per 1,000 PM (1.1 - 11.7) | 1 / 891, 2.2 per 1,000 PM (0.0 - 6.8) | 0.45 | 24 / 777, 19.0 per 1,000 PM (9.9 - 31.1) | 7 / 823, 2.4 per 1,000 PM (0.0 - 7.4) | <0.01** |
| Musculoskeletal | 0 / 783, 0.0 per 1,000 PM (0.0 - 0.0) | 5 / 745, 5.4 per 1,000 PM (1.3 - 13.8) | 0.11 | 0 / 900, 0.0 per 1,000 PM (0.0 - 0.0) | 0 / 900, 1.1 per 1,000 PM (0.0 - 3.4) | 1 | 7 / 813, 7.2 per 1,000 PM (1.2 - 14.9) | 0 / 840, 1.2 per 1,000 PM (0.0 - 3.6) | 0.12 |
| Neuro-cognitive | 5 / 770, 3.9 per 1,000 PM (0.0 - 10.4) | 18 / 730, 15.7 per 1,000 PM (6.5 - 27.7) | 0.06 | 2 / 901, 4.5 per 1,000 PM (1.1 - 9.1) | 2 / 893, 4.5 per 1,000 PM (1.1 - 10.4) | 0.72 | 7 / 826, 16.2 per 1,000 PM (7.3 - 27.9) | 1 / 830, 6.0 per 1,000 PM (1.2 - 12.1) | 0.07 |
| Respiratory | 0 / 783, 0.0 per 1,000 PM (0.0 - 0.0) | 4 / 774, 5.2 per 1,000 PM (0.0 - 12.3) | 0.12 | 0 / 900, 0.0 per 1,000 PM (0.0 - 0.0) | 0 / 900, 0.0 per 1,000 PM (0.0 - 3.4) | 1 | 5 / 819, 6.1 per 1,000 PM (1.2 - 13.6) | 0 / 837, 0.0 per 1,000 PM (0.0 - 3.6) | 0.12 |
| Sensory | 2 / 780, 1.3 per 1,000 PM (0.0 - 3.9) | 6 / 678, 13.2 per 1,000 PM (4.3 - 25.4) | 0.01* | 1 / 891, 1.1 per 1,000 PM (0.0 - 3.4) | 0 / 900, 0.0 per 1,000 PM (0.0 - 0.0) | 1 | 15 / 795, 11.1 per 1,000 PM (3.6 - 21.7) | 0 / 840, 0.0 per 1,000 PM (0.0 - 0.0) | <0.01** |
| Sleep | 1 / 793, 6.4 per 1,000 PM (1.3 - 13.2) | 18 / 726, 23.4 per 1,000 PM (11.9 - 38.5) | 0.01* | 3 / 890, 5.6 per 1,000 PM (1.1 - 11.4) | 3 / 894, 3.3 per 1,000 PM (0.0 - 7.8) | 0.62 | 15 / 798, 24.5 per 1,000 PM (13.6 - 38.8) | 3 / 828, 3.6 per 1,000 PM (0.0 - 8.4) | <0.01** |
| **Prevalence of wellbeing deterioration according to VAS (event rate, %)** | | | | | | | | | |
| Behavior | 16 / 250, 7.3% (4.1% - 11.1%) | 31 / 248, 9.7% (6.0% - 14.1%) | 0.35 | 22 / 279, 7.0% (4.0% - 10.3%) | 9 / 277, 4.0% (1.8% - 6.8%) | 0.15 | 37 / 265, 10.4% (6.7% - 14.9%) | 10 / 247, 4.2% (1.6% - 7.1%) | <0.01** |
| Communication | 6 / 224, 1.8% (0.4% - 3.8%) | 9 / 233, 3.0% (0.9% - 6.1%) | 0.42 | 3 / 251, 1.3% (0.0% - 3.2%) | 0 / 250, 0.8% (0.0% - 2.5%) | 0.54 | 12 / 255, 3.6% (1.2% - 6.5%) | 4 / 224, 1.2% (0.0% - 2.7%) | 0.11 |
| Emotional | 22 / 237, 8.1% (4.6% - 12.1%) | 19 / 252, 12.2% (8.0% - 17.2%) | 0.15 | 22 / 281, 7.8% (4.7% - 11.4%) | 13 / 268, 5.5% (2.9% - 8.8%) | 0.31 | 45 / 267, 12.9% (8.7% - 17.5%) | 16 / 259, 5.8% (3.1% - 9.2%) | <0.01** |
| Fatigue | 11 / 246, 7.4% (4.1% - 11.2%) | 41 / 259, 14.9% (10.4% - 19.8%) | <0.01** | 15 / 272, 6.7% (3.9% - 10.1%) | 17 / 287, 6.5% (3.6% - 9.9%) | 0.53 | 40 / 276, 16.0% (11.4% - 21.1%) | 19 / 269, 7.1% (4.1% - 10.6%) | <0.01** |
| Friends personally | 3 / 169, 3.4% (1.1% - 6.5%) | 10 / 212, 5.5% (2.3% - 9.5%) | 0.36 | 5 / 195, 2.6% (0.5% - 5.3%) | 1 / 210, 0.9% (0.0% - 2.8%) | 0.26 | 7 / 227, 5.4% (2.2% - 9.4%) | 9 / 211, 1.4% (0.0% - 3.3%) | 0.03* |
| Friends remotely | 0 / 150, 0.7% (0.0% - 2.2%) | 0 / 208, 0.5% (0.0% - 2.4%) | 0.65 | 0 / 157, 0.6% (0.0% - 2.0%) | 2 / 183, 1.0% (0.0% - 2.6%) | 0.67 | 2 / 229, 0.9% (0.0% - 2.6%) | 2 / 189, 1.0% (0.0% - 2.7%) | 0.61 |
| Physical activity | 13 / 249, 2.7% (0.8% - 5.2%) | 24 / 244, 9.3% (5.5% - 13.8%) | <0.01** | 6 / 287, 2.5% (1.0% - 4.8%) | 18 / 292, 5.8% (3.4% - 9.0%) | 0.07 | 26 / 265, 10.0% (6.1% - 14.3%) | 18 / 268, 6.6% (3.7% - 9.9%) | 0.18 |
| Relations | 1 / 215, 0.9% (0.0% - 2.4%) | 13 / 252, 4.9% (2.4% - 8.4%) | 0.01* | 2 / 242, 0.4% (0.0% - 2.0%) | 1 / 245, 0.8% (0.0% - 2.4%) | 0.62 | 17 / 264, 5.7% (2.7% - 9.2%) | 1 / 247, 0.8% (0.0% - 2.5%) | <0.01** |
| School attendance | 12 / 165, 8.6% (4.3% - 13.6%) | 20 / 178, 11.3% (6.4% - 17.1%) | 0.39 | 13 / 172, 9.0% (5.1% - 13.6%) | 6 / 198, 3.8% (1.4% - 7.2%) | 0.05* | 23 / 198, 11.5% (6.9% - 16.8%) | 3 / 183, 4.5% (1.6% - 8.1%) | 0.01* |
| Screentime for education | 0 / 131, 0.0% (0.0% - 0.0%) | 0 / 200, 0.0% (0.0% - 1.9%) | 1 | 0 / 156, 0.0% (0.0% - 0.0%) | 7 / 198, 2.8% (0.5% - 5.7%) | 0.06 | 0 / 234, 0.4% (0.0% - 2.2%) | 6 / 186, 2.7% (0.5% - 5.6%) | 0.09 |
| Screentime for leisure | 2 / 144, 0.7% (0.0% - 2.3%) | 4 / 216, 0.9% (0.0% - 2.8%) | 0.65 | 0 / 174, 0.6% (0.0% - 2.0%) | 2 / 190, 2.5% (0.5% - 5.1%) | 0.24 | 1 / 234, 0.9% (0.0% - 2.6%) | 2 / 198, 2.5% (0.5% - 5.2%) | 0.25 |
| Sleep | 21 / 253, 8.5% (5.1% - 12.4%) | 27 / 255, 11.9% (7.8% - 16.6%) | 0.22 | 23 / 286, 7.6% (4.7% - 11.0%) | 14 / 298, 5.4% (3.0% - 8.3%) | 0.29 | 35 / 275, 12.2% (8.1% - 16.8%) | 13 / 273, 5.4% (2.9% - 8.5%) | <0.01** |
| Time outdoors | 4 / 195, 2.1% (0.5% - 4.6%) | 12 / 237, 5.3% (2.5% - 8.9%) | 0.11 | 3 / 225, 2.2% (0.5% - 4.4%) | 13 / 278, 3.8% (1.5% - 6.5%) | 0.35 | 19 / 257, 5.6% (2.7% - 9.1%) | 7 / 253, 5.0% (2.4% - 8.1%) | 0.5 |

## Figure S1. Time of study participants hospital admission and their chronological correspondence to COVID-19 variant dominance in Moscow city.


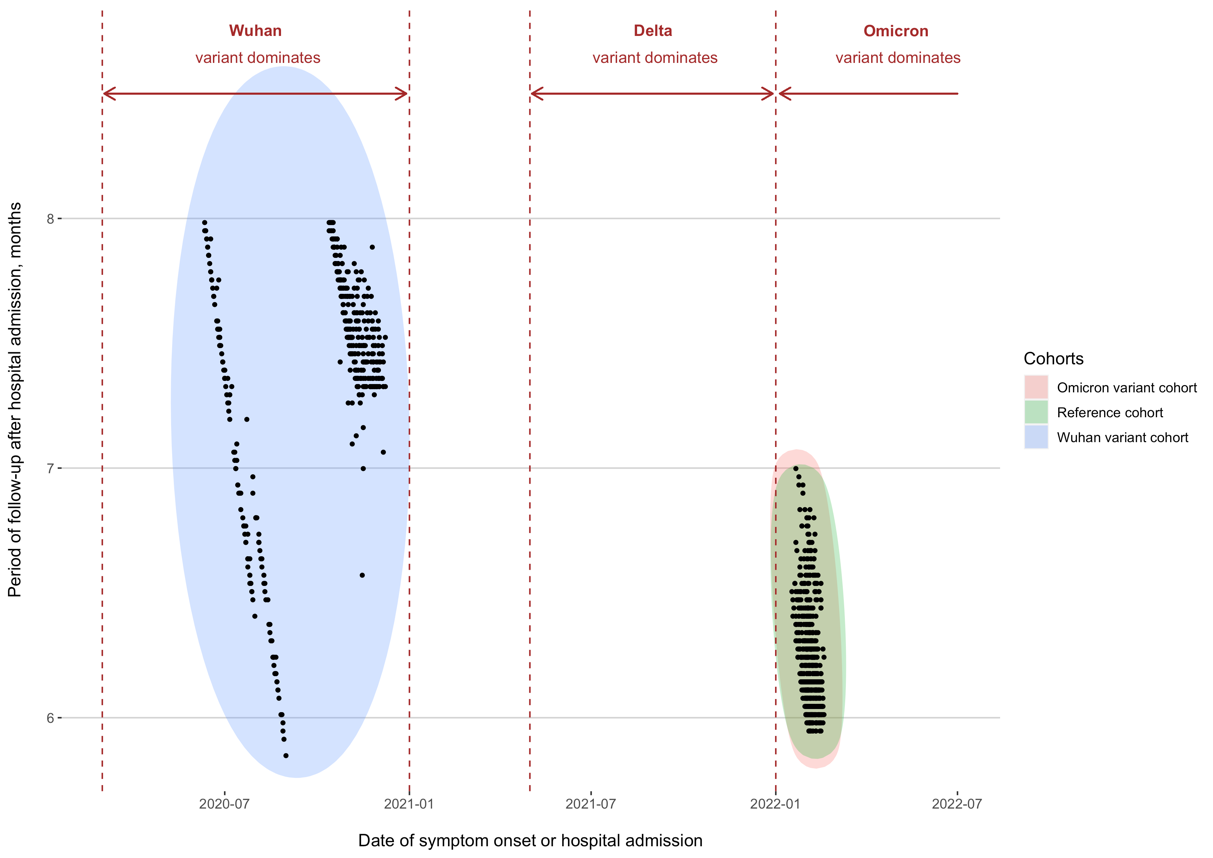


Figure S2. Incidence of post‐COVID‐19 condition manifestations in matched exposed and reference groups, sensitivity analysis.
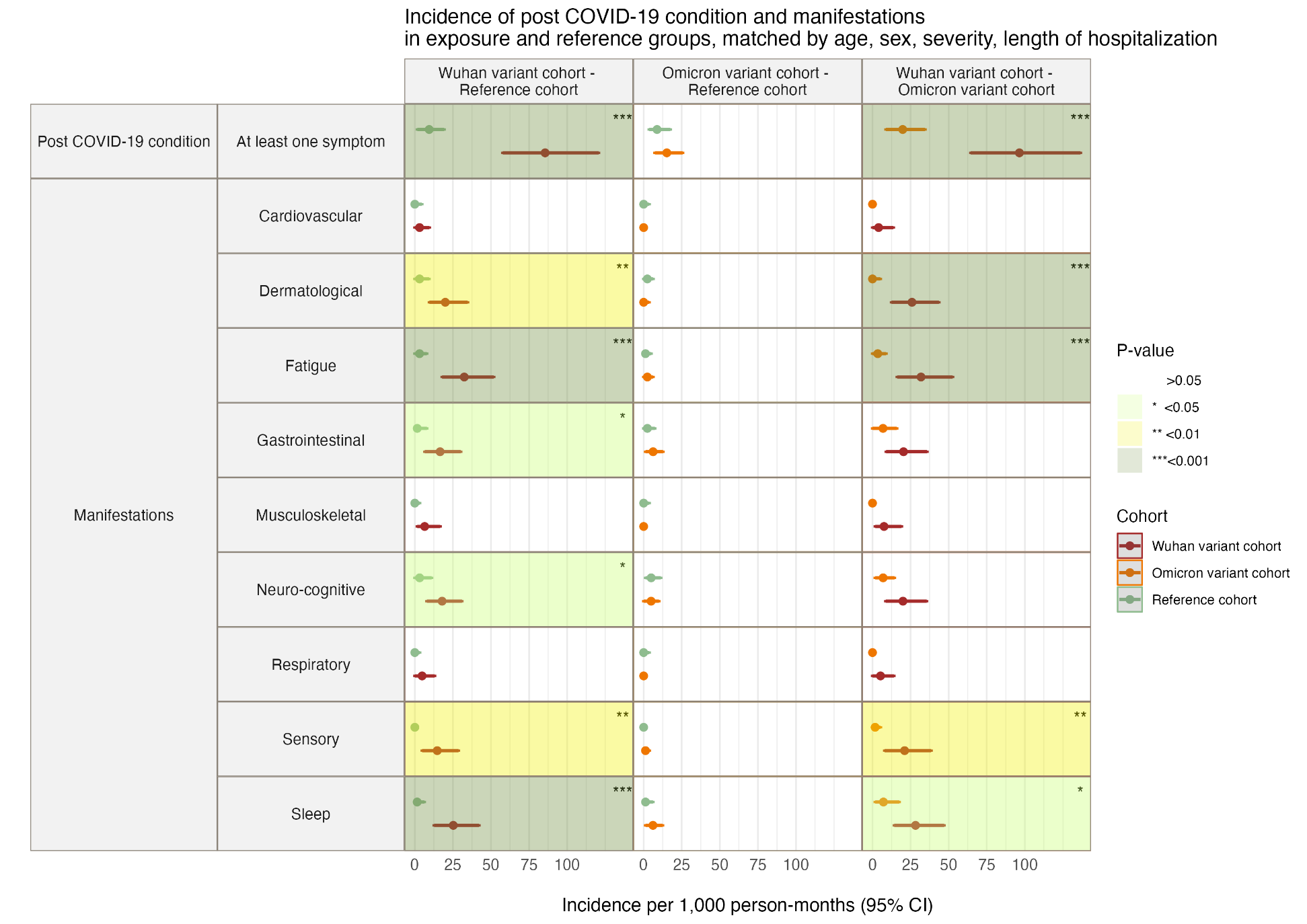


**Figure S3.** Rates of events for incomplete recovery and emotional behavioural changes in matched exposed and reference groups, sensitivity analysis.


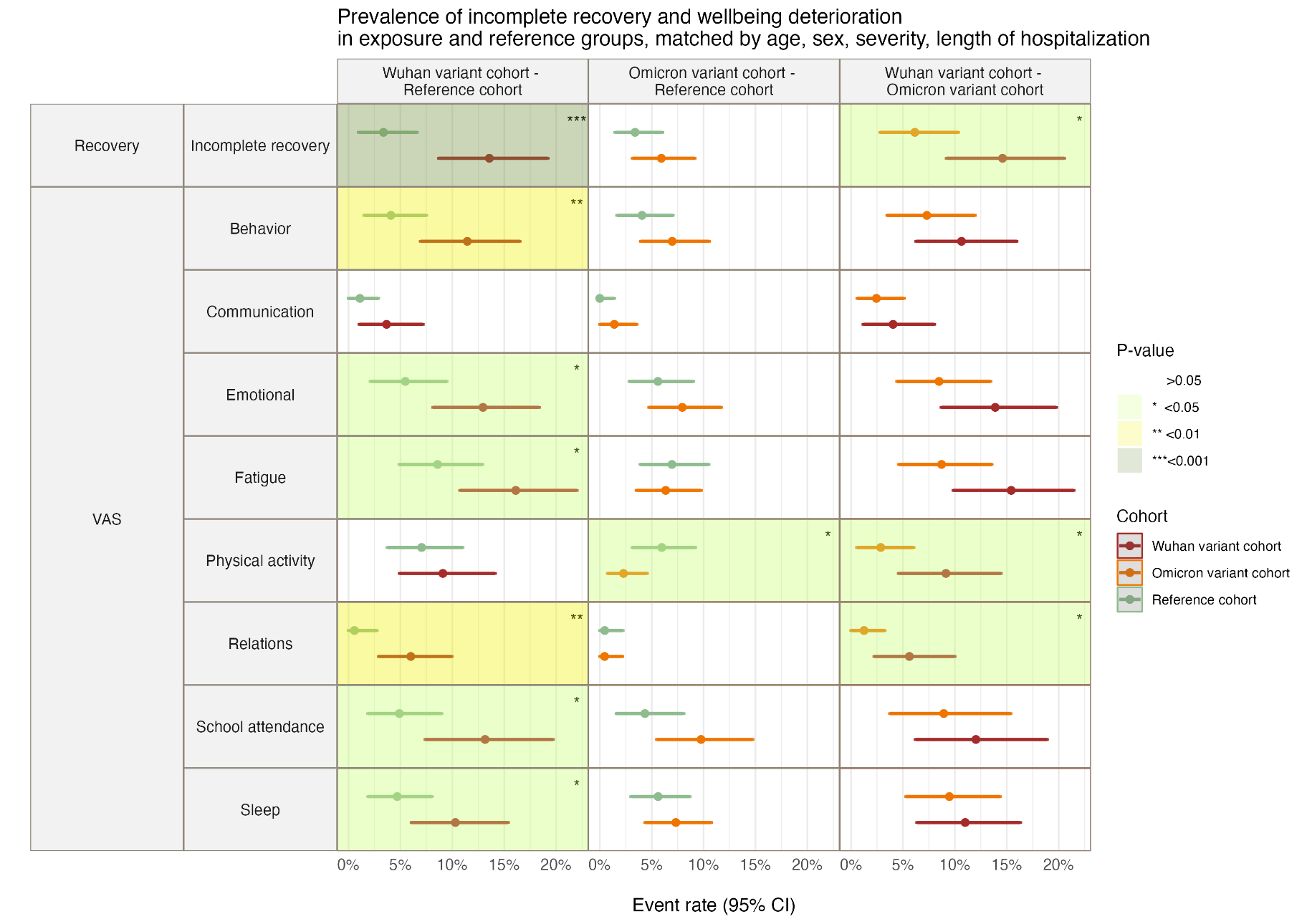

Supplement: Supplementary file 1 — Additional file 1: Table S1. Criteria for hospital admission as per local clinical guidelines. Table S2. Demographic characteristics of study participants—initial and matched cases, sensitivity analysis. Table S3. Incidence and prevalence of Post COVID-19 manifestations. Figure S1. Time of study participants hospital admission and their chronological correspondence to COVID-19 variant dominance in Moscow city. Figure S2. Incidence of post-COVID-19 condition manifestations in matched exposed and reference groups, sensitivity analysis. Figure S3. Rates of events for incomplete recovery and emotional behavioural changes in matched exposed and reference groups, sensitivity analysis. [file 12916_2023_3221_MOESM1_ESM.docx]
